# Modified SJH alleviates FFAs-induced hepatic steatosis through leptin signaling pathways

**DOI:** 10.1038/srep45425

**Published:** 2017-03-30

**Authors:** Dong-Woo Lim, Shambhunath Bose, Jing-Hua Wang, Han Seok Choi, Young-Mi Kim, Young-Won Chin, Song-Hee Jeon, Jai-Eun Kim, Hojun Kim

**Affiliations:** 1 Department of Pathology, College of Oriental Medicine, Dongguk University, Goyang, Republic of Korea; 2 Departments of Rehabilitation Medicine, College of Oriental Medicine, Dongguk University, Goyang, Republic of Korea; 3 Applied Surface Technology Inc., 11th Floor, Bldg. A, Advance Institutes of Convergence Technology, Suwon, 16229, Republic of Korea; 4 Division of Endocrinology and Metabolism, Department of Internal Medicine, Dongguk University Ilsan Hospital, Goyang, Republic of Korea; 5 College of Pharmacy and Integrated Research Institute for Drug Development, Dongguk University-Seoul, Goyang, Republic of Korea; 6 Research Institute of Biotechnology, Dongguk University, Goyang, Republic of Korea

## Abstract

Samjunghwan (SJH) is an herbal formula used in traditional Korean medicine. This prescription has long been used in treatment of aging and lifestyle diseases. The current study showed the effect and mechanisms of anti-hepatic steatosis action of modified SJH (mSJH) *in vitro* and *in vivo*. Treatment with mSJH resulted in significantly decreased intracellular lipid accumulation in steatosis-induced cells. Furthermore, mSJH triggered the phosphorylation of AMP-activated protein kinase and *acetyl-CoA carboxylase* as well as increased the expression of leptin at both protein and gene levels. In addition, C57BL6 mice fed high-fat diet (HFD) showed significant improvements in body, liver weights and fat weights; and serum, hepatic and fecal lipid parameters in response to the treatment with mSJH. Furthermore, mSJH showed favorable effects on the hepatic expression of several genes related to lipid metabolism. Betaine, one of constituents of mSJH exerted fundamental beneficial impact on FFAs-induced cells. However, the beneficial effects of mSJH were diminished upon blocking of leptin signaling by dexamethasone, suggesting the leptin signaling as a key component in mSJH-mediated modulation of lipid homeostasis. Our results suggest that mSJH exerts an anti-hepatic steatosis effect via activation of leptin and associated signaling cascades related to lipid metabolism.

Hepatic steatosis (also termed as fatty liver, HS) represents a pathological state characterized by excessive intracellular accumulation of lipids, especially triglycerides and other fatty acids in the liver resulting from an imbalance between the uptake of fat and its oxidation and export. Further studies have reported that overwhelming concentration of free fatty acids (FFAs) induces inflammation in liver tissue^1^ and causes disruption of endoplasmic reticulum (ER) homeostasis, often termed as ER stress, which can trigger HS^2^. HS can progress to non-alcoholic steatohepatitis (NASH) or non-alcoholic fatty liver disease (NAFLD) and finally to liver cirrhosis^3^. HS distorts the structure of the liver and impairs its function, but usually without accompanying symptoms except fatigue and discomfort in the abdominal region^4^. The incidence of HS has shown an alarming increase each year and is currently recognized as the most common liver disease worldwide^5^. Clinical studies have indicated that HS has a significant association with metabolic disease including obesity, hyperlipidemia, and type 2 diabetes^6^. Accumulating evidence suggests that NAFLD can predict the development of diabetes and vice versa and that each condition serves as a progression factor for the other^7^.

Adipokines include adiponectin, resistin, adipsin, adiponutrin, retinol-binding protein (RBP), insulin-like growth factor-1 (IGF-1) etc^8^. Emerging evidence indicates that the levels of some adipokines correlate with specific metabolic states can directly influence the metabolic homeostasis of the system^9^. Several clinical observations reveal a link between adiponectin level and obesity-related metabolic dysfunction^10^, and association of dysregulation of adipokines with obesity, type 2 diabetes, and hypertension^9^ has been reported.

Leptin, a major adipokine secreted primarily from maturated adipocytes, plays a key role in regulation of food intake, energy expenditure, and neuroendocrine function^11^. This protein exerts its effects by interacting with the long form of the leptin receptor, Ob-Rb. Several lines of evidence suggest that leptin may be an important factor linking obesity, metabolic syndrome, and cardiovascular disorders^12^. This adipokine plays key roles in lipid homeostasis by modulating p-*acetyl-CoA carboxylase* (ACC)/p-AMP-activated protein kinase (AMPK) signaling pathways^13^. Leptin has also been shown to inhibit insulin stimulated fatty acid uptake in differentiated 3T3-L1 murine adipocyte^14^. It reduces food intake and increases energy consumption by acting on specific hypothalamic nuclei, inducing anorexigenic factors and counteracting the effect of orexigenic neuropeptides^10^. Furthermore, based on emerging reports, the attenuating effect of leptin on HS has become more evident^15^.

SJH (*Samjunghwan*), a popular herbal medicinal formula in Korea, consists of three medicinal herbs, Atractylodes rhizome, Lycii Radicis Cortex, and Mori Fructus. This formulation has long been used as an anti-aging therapeutic agent^16^. Recent *in vitro* and *in vivo* studies have reported a number of beneficial effects of SJH or its fermented products including neuroprotective^17^, anti-oxidant^18^, and anti-obesity^19^ properties, and several studies have shown a beneficial effect of *Mori fructus*, the major constituent of SJH, on obesity and related complications^20^,^21^. *In vitro* and *in vivo* studies have found that *Mori fructus* exerts hypolipidemic effects through the modulation of lipid homeostasis^22^.

In the current study, we prepared a modified SJH (mSJH) formulation by altering the herbal composition and extraction method of conventional SJH with the aim of improving its beneficial pharmacological properties. Using a human hepatocellular *in vitro*^23^ and a high-fat diet (HFD)-induced mouse *in vivo* steatosis models with advanced molecular tools for gene and protein expression analyses, we attempted to determine whether mSJH could alleviate HS, and if so what are the probable underlying molecular mechanisms. Our results indicate that mSJH prevents hepatic steatosis by increasing leptin expression level and thereby activating lipid metabolism pathways.

## Results

### UPLC-MS analyses of mSJH and its components

For profiling the major molecular ingredients of the proposed herbal preparation, UPLC-MS analyses of mSJH and its components, Morus Fructus, Lycii Radicis Cortex, and Atractylodes Rhizoma were performed (Fig. S1). By comparing the protonated molecular ion peaks appearing in UPLC-MS chromatograms with the molecular weights of the known compounds present in these three plant materials, cyanidin-3-*O*-rutinoside (Morus Fructus), betaine (Lycii Radicis Cortex), kukoamine A (Lycii Radicis Cortex), atractylodin (Atractylodes Rhizoma), and atractylenolide II (Atractylodes Rhizoma) were tentatively identified.

### mSJH did not affect HepG2 cell proliferation at experimental concentrations.

Prior to examining the anti-obesity impact of mSJH extract, its cytotoxicity was evaluated in human hepatoma HepG2 cells used as a model in our study. No cytotoxicity was produced by exposure of the cells to mSJH alone up to a concentration of 400 μg/ml for 24 h (Fig. S2A). Also, no adverse effect of mSJH was observed on the cells when applied at concentrations ranging from 50–400 μg/ml in combination with 2 mM FFAs (Fig. S2B).

### Antioxidant activities and inhibitory effect of mSJH on nitric oxide (NO) production

Antioxidant effect of mSJH was examined in terms of its 2,2-diphenyl-1-picrylhydrazyl (DPPH) free radical scavenging activity using butylated hydroxytoluene as a positive control, and the impact of mSJH on H_2_O_2_-induced intracellular reactive oxygen species (ROS) production was also evaluated. Our results demonstrated an inhibitory activity of mSJH on DPPH, causing 22% suppression of this radical at 250 μg/ml concentration (Fig. S3A). In parallel, significant inhibition in the intracellular ROS generation was also observed in H_2_O_2_-induced cells co-treated with mSJH at 100, 200, or 400 μg/ml (Fig. S3B). Results of further analyses of the composition of mSJH showed total phenolic, flavonoid and tannin contents of 17.20, 26.51, and 13.84 mg/g, respectively (Fig. S3C).

An exposure of cells to 2 mM FFAs for 48 h resulted in a marked increase in the NO production. Co-treatment of cells with mSJH caused significant inhibition of FFAs-induced NO production in a concentration-dependent manner (Fig. S3D).

### mSJH alleviated FFAs-induced lipid accumulation in the cell model of hepatic steatosis

**Figure 1:**
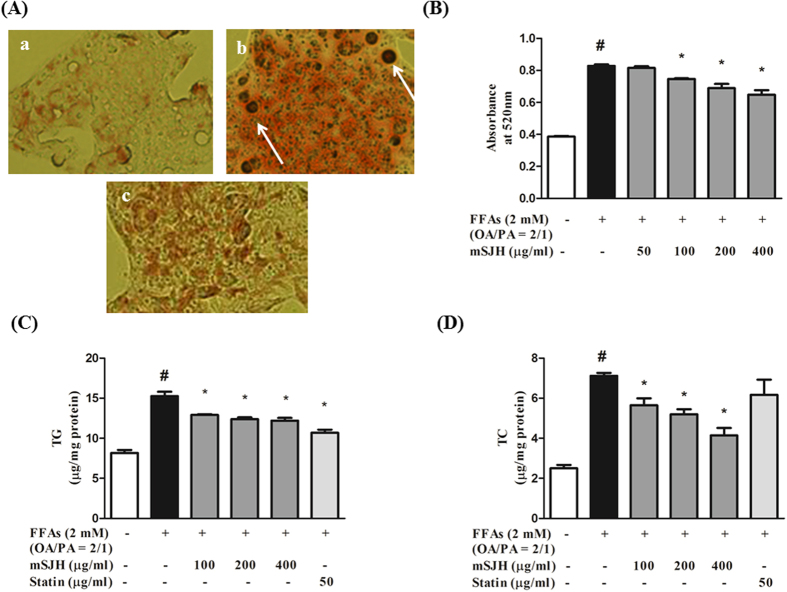
Microscopic images showing the effect of mSJH on the intracellular lipid accumulation in FFAs-treated HepG2 cells in terms of their Oil Red O staining (**A**). The cells were uninduced (a), FFAs-induced (b), or exposed to FFAs in combination with 400 μg/ml mSJH (c). Major droplets of intracellular lipids are indicated by arrows. Spectrophotometric-based quantitative measurement of the Oil Red O staining of cells subjected to different experimental conditions is shown (**B**). Evaluation of the effect of mSJH on the intracellular levels of TG (**C**) and TC (**D**) in FFAs-induced hepatic steatosis model of HepG2 cells. Data are expressed as mean ± SD (n = 3). ^#^p < 0.05 vs normal and *p < 0.05 vs FFAs treatment.

Treatment of HepG2 cells with FFAs resulted in a significant increase in lipid accumulation (114%) as evident by Oil Red O staining (Fig. 1A,B). Co-treatment of FFAs-induced cells with mSJH resulted in significantly reduced intracellular lipid deposition in a concentration-dependent manner at 100, 200, and 400 μg/ml. The lipid level of the FFAs-treated cells was decreased by 22% when co-exposed to 400 μg/ml mSJH.

In keeping with the augmented intracellular lipid accumulation, FFAs treatment increased the levels of TG and TC in the cells (Fig. 1C,D). Exposure of FFAs-induced cells to mSJH resulted in significantly reduced levels of both lipids in a concentration-dependent manner.

### mSJH modulates gene expression related to lipid metabolism

**Figure 2:**
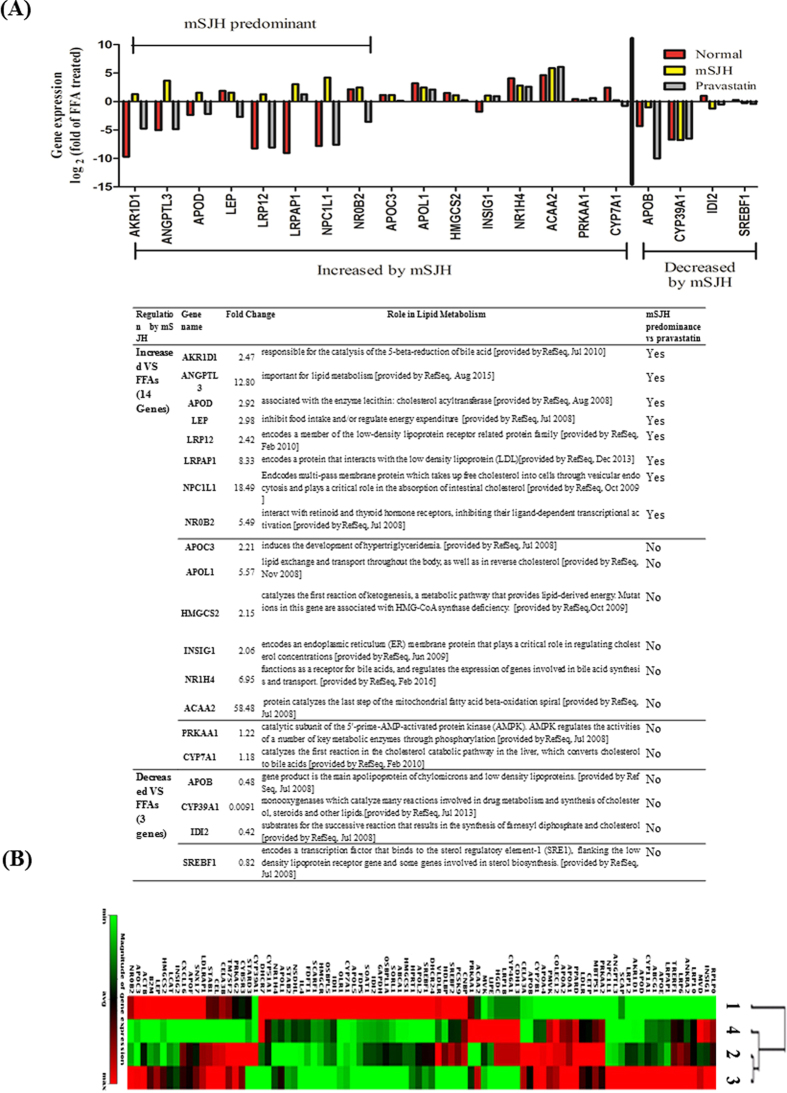
Impact of mSJH on the expression profile of genes related with lipoprotein signaling and cholesterol metabolism in FFAs-induced hepatic steatosis model of HepG2 cells as determined by PCR array. The bar diagram denotes the level of expression of genes of normal, FFAs + mSJH and FFAs + pravastatin-treated cells normalized with that of FFAs-treated cells (log_2_x) (**A**). The figure represents only those genes that exhibit marked differences in their expression among the experimental groups. Heatmap of PCR array data representing clustering of expressed genes (**B**); 1-Normal, 2-FFAs-induced, 3-FFAs + mSJH treated and 4 -FFAs + pravastatin treated.

To understand the molecular mechanism of action of mSJH against FFAs-induced lipid accumulation in HepG2 cells, PCR microarray was performed for analysis of the expression of vital genes involved in lipoprotein signaling and cholesterol metabolism (Fig. 2A,B). Our results based on heat map combined with hierarchical clustering analysis showed that the clustering of these genes in the normal group is separated from the other three groups, albeit mostly relevant to the FFAs + pravastatin group while the gene expression profile of the FFAs + mSJH group is similar to that of the FFAs-induced group.

Among the 89 genes analyzed by PCR array, 14 genes were upregulated (≧2 times) and 3 genes were downregulated (≦0.5 times) in the FFAs + mSJH-treated group compared with the FFAs-induced group. Among these upregulated genes, 8 genes were categorized as the SJH- predominant group (≧2 times higher expression than FFAs + pravastatin group) where the level of expressions of leptin (LEP) and small heterodimer partner (NR0B2) genes were recovered to almost that of the normal group.

### mSJH regulates the key components of lipogenesis and lipid metabolic pathways

**Figure 3:**
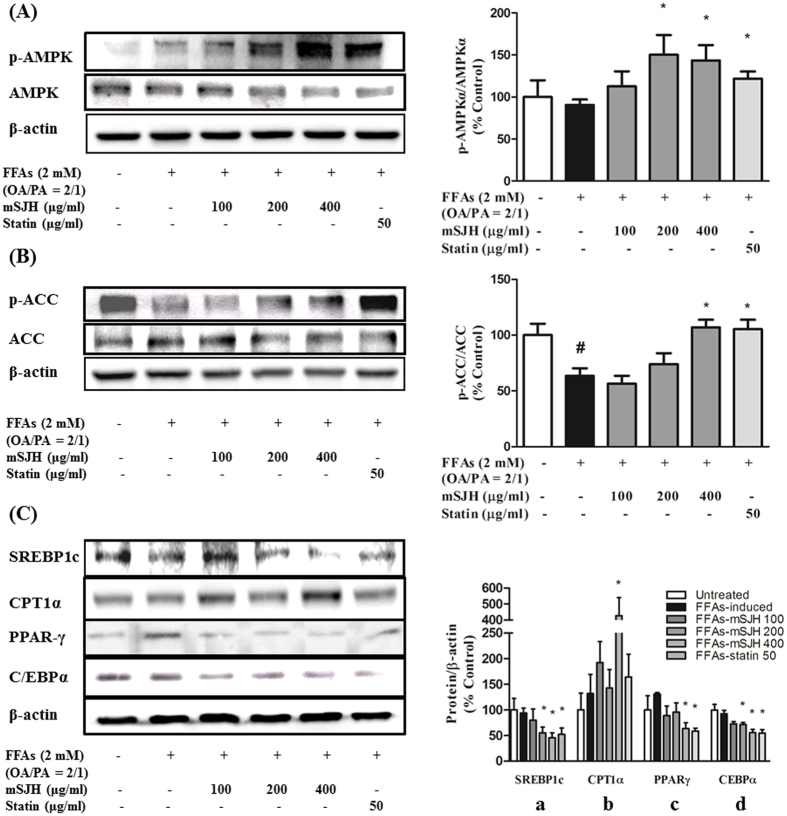
Western blot analysis showing the impact of mSJH on the phosphorylation of AMPKα (**A**) and ACC (**B**) in FFAs-induced HepG2 cells. Representative blots from at least three individual studies are depicted (left panel). The density of the bands in blots was measured and normalized to the quantity of non-phosphorylated form of AMPK or ACC (right panel). Data are expressed as mean ± SD (n = 3). Western blot analysis showing the effect of mSJH on the expression of SREBP1c, CPT1α, PPAR-γ, and C/EBPα proteins in FFAs-treated HepG2 cells (**C**). Representative blots from at least three individual studies are depicted (left panel). The density of the bands in blots was measured and normalized to the quantity of β-actin (right panel). Data are expressed as mean ± SD (n = 3). ^#^p < 0.05 vs normal and *p < 0.05 vs FFAs treatment.

The PCR array data showed that co-exposure of FFAs-treated cells to mSJH and pravastatin resulted in increased expression of the AMPKa1 (PRKAA1) gene by 22% and 54%, respectively, and decreased expression of SREBP (SREBF1) by 18% and 25%, respectively (Fig. 2A). Results of Western blotting analyses showed that co-treatment of FFAs-induced cells with mSJH result in significantly increased phosphorylation of AMPK by 66% and 58% at 200 and 400 μg/ml concentrations, respectively (Fig. 3A) and phosphorylation of ACC in FFAs-treated cells was significantly increased by 68% and 66% when exposed to 400 μg/ml of mSJH and 50 μg/ml of pravastatin, respectively (Fig. 3B).

Protein expression of both SREBP1c and CCAAT/Enhancer Binding Protein α (C/EBPα) in FFAs-induced cells were significantly inhibited by mSJH at 200 and 400 μg/ml concentrations and 50 μg/ml of pravastatin (Fig. 3C). The level of carnitine palmitoyltransferase-1α (CPT1α) in FFAs-treated cells remained unchanged when co-treated with 100 and 200 μg/ml of mSJH and 50 μg/ml of pravastatin, but significantly increased when co-exposed to 400 μg/ml of mSJH (Fig. 3C) while the expression of peroxisome proliferator-activated receptor (PPAR)-γ was significantly decreased in FFAs-induced cells by co-treatment with 400 μg/ml of mSJH and 50 μg/ml of pravastatin (Fig. 3C).

### mSJH modulates the gene and protein expression of leptin

The PCR array data showed that expression of leptin gene was decreased by 73% in FFAs-induced cells (Fig. 2A). However, co-exposure of FFAs-treated cells to mSJH recovered the expression level of leptin to almost the normal level (198% increase vs FFAs group). Accordingly, real-time PCR and Western blotting were performed in order to further confirm this finding on leptin gene expression.

**Figure 4:**
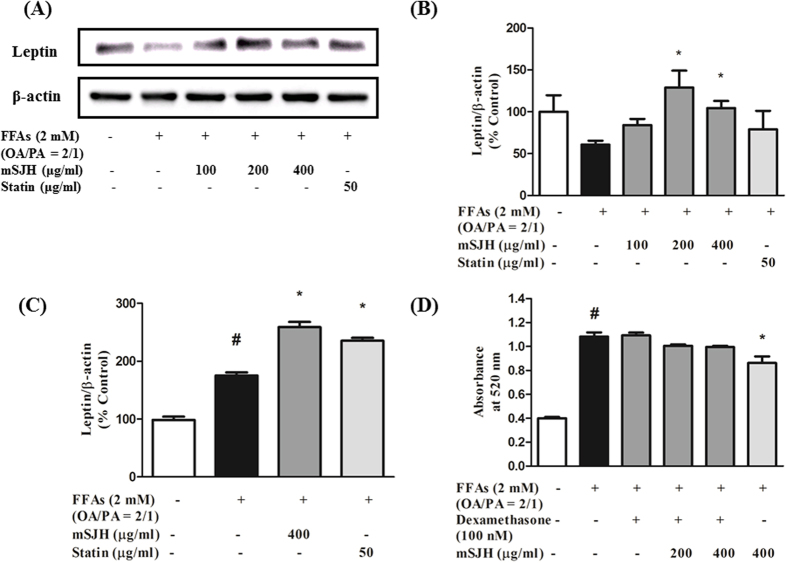
Quantitative real-time PCR and Western blot analyses showing the impact of mSJH on the gene and protein expression of leptin in FFAs-induced HepG2 cells (**A**–**C**). Representative blot from at least three individual western blot studies is depicted (**A**). The density of the bands in blots was measured and normalized to the quantity of β-actin (**B**). The gene expression of leptin in relation to that of β-actin is shown (**C**). Effect of impairment of leptin signaling on the mSJH-mediated inhibition of intracellular lipid accumulation in FFAs-treated HepG2 cells (**D**). Data are expressed as mean ± SD (n = 3). ^#^p < 0.05 vs normal and *p < 0.05 vs FFAs treatment.

The quantitative real-time PCR data showed that co-treatment of FFAs-induced cells with mSJH at 400 μg/ml resulted in significantly increased expression of the leptin gene (Fig. 4C). This was further supported by the Western blotting data which showed that the protein expression of leptin in FFAs-treated cells was significantly augmented upon co-treatment with mSJH at 200 and 400 μg/ml concentrations (Fig. 4B).

### mSJH exerts its effectiveness through leptin signaling pathway

Western blot analysis demonstrated marked reduction of leptin level in HepG2 cells in response to the treatment with dexamethasone at 3, 6, 12, 24 h (corresponding to 83%, 80%, 86%, and 41% decreases, respectively vs. 0 h) (Fig. S4A). Additionally, pretreatment of cells with dexamethasone (30 min) strongly inhibited mSJH-induced expression of leptin (Fig. S4B). In parallel, the extent of mSJH-induced phosphorylation of AMPK in the cells was decreased abruptly upon co-exposure to dexamethasone (Fig. S4C). Furthermore, Oil Red O staining results revealed that the inhibitory effect of mSJH on lipid accumulation in cells was diminished in response to dexamethasone pretreatment (30 min) (Fig. 4D).

### mSJH attenuates weights of body, liver and intestinal fat, and improved vital hepatic and serum lipid parameters and enzymatic activities in HFD-fed mice

**Figure 5:**
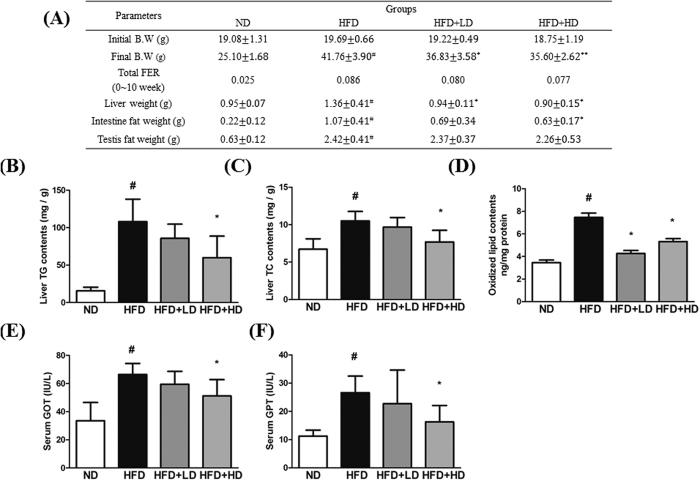
Effect of mSJH treatment on the body weight changes, food efficiency ratio, and liver and fat tissue weights in mice fed high-fat diet (**A**). Impact of mSJH treatment on the hepatic lipid profile (**B**,**C**) and oxidized lipid content (**D**), and serum GOT (**E**) and GPT (**F**) levels in mice fed high-fat diet. Data are expressed as Mean ± SD (n = 8). ^#^p < 0.05 vs ND group and *p < 0.05 vs HFD group.

As expected, at the termination of experimental duration (10th week), the body weight of HFD group was significantly higher compared to that of ND group. The liver weight as well as the intestinal and testicular fat weights was also significantly greater in HFD-fed mice vs ND-fed mice. However, an exposure of HFD-fed animals to either lower dose (LD group) or higher dose (HD group) of mSJH significantly decreased both the body and liver weights. The total food efficiency ratio (FER) was also higher in HFD-group compared to ND group. Treatment of HFD group with either higher or lower doses of mSJH exerted a negative impact on the FER as well as intestinal and testicular fat weights (Fig. 5A).

**Figure 6:**
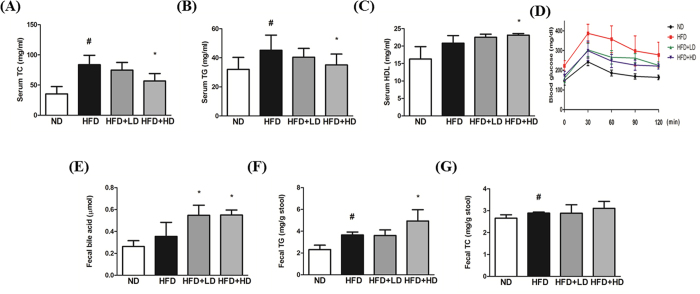
Effect of mSJH treatment on the serum TC, TG, and high-density lipoprotein levels in mice fed high-fat diet (**A**–**C**, respectively). Impact of mSJH on the blood glucose level in OGTT at the indicated time-intervals after high-glucose load in mice fed high-fat diet (**D**). Effect of mSJH on the fecal bile acid, TG, TC levels in mice fed high-fat diet E, F and G, respectively). Data are expressed as Mean ± SD (n = 8). ^#^p < 0.05 vs ND group and *p < 0.05 vs HFD group.

Both hepatic and serum TG and TC levels as well as serum GOT and GPT activities were significantly higher in HFD group compared to ND group. However, an exposure of HFD-fed animals to the higher dose of mSJH significantly decreased all of these parameters (Figs 5B,C,E,F and 6A,B). The oxidized lipid content of the liver was significantly greater in HFD group vs ND group. Treatment of HFD-fed animals with both higher and lower doses of mSJH significantly decreased the hepatic oxidized lipid content (Fig. 5D). While, the serum HDL level of HFD-fed mice increased significantly in response to the treatment with higher dose of mSJH (Fig. 6C).

### mSJH alleviates blood glucose level in HFD-fed mice

Our OGTT test demonstrated significantly higher levels of blood glucose in HFD group compared to ND group at every time points (0, 30, 60, 90 min) of measurement (Fig. 6D). However, the glucose clearance rate was significantly improved in HFD-fed animals at all measurement time points in response to the treatment with both lower and higher doses of mSJH (p < 0.05, 30, 60, 90, 120 min; except for the lower dose at 90 min).

### Effect of mSJH on fecal lipid contents in HFD-fed mice

Treatment with both lower and higher doses of mSJH significantly elevated the fecal bile acid content in HFD group. While the fecal TG content of HFD-fed animals was significantly increased upon an exposure to higher dose of mSJH (Fig. 6F). However, both the lower and higher doses of mSJH did not produce any significant effect on the fecal TC content of HFD group (Fig. 6G).

### Histological analysis of mice liver

**Figure 7:**
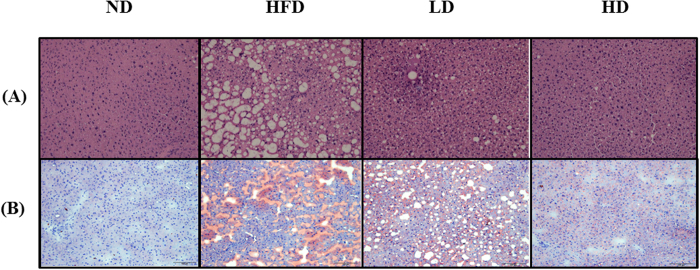
Effect of lower and higher doses of mSJH (LD and HD, respectively) on the liver histology of mice fed high-fat diet. The images of hematoxylin-eosin (H&E)- and Oil Red O-stained tissue sections of liver (**A** and **B**, respectively) were captured by microscopy at 200X magnification. The nearby area of central vein in the images is projected (arrow head).

Hematoxylin & Eosin staining of the liver tissue sections revealed extensive hepatic deposition of fat droplets in HFD group in the form of unstained microvacuoles. This was further supported by the Oil Red O staining of the tissue sections. However, treatment of HFD-fed animals with both lower and higher doses of mSJH markedly reduced the hepatic fat accumulation (Fig. 7A).

### mSJH modulates the expression of genes or proteins related to lipid metabolism in mice liver

**Figure 8:**
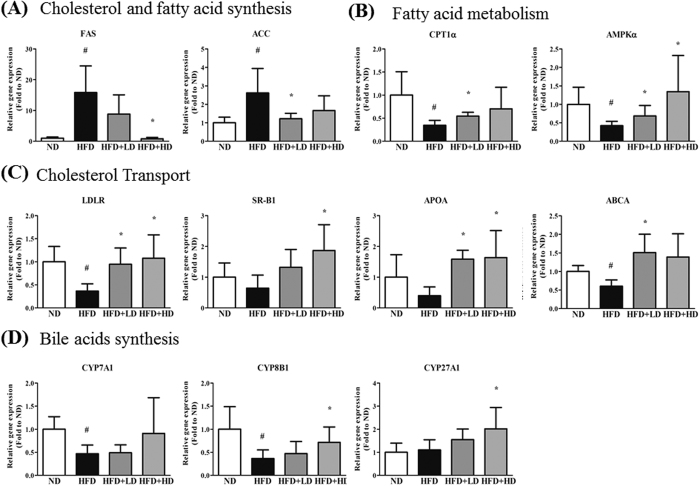
Quantitative real-time PCR showing the impact of lower and higher doses of mSJH (LD and HD, respectively) on the hepatic expression of genes involved in cholesterol and fatty acid synthesis (**A**), fatty acid metabolism (**B**), cholesterol transport (**C**), and bile acid synthesis (**D**) in mice fed high-fat diet. The gene expression of a reference protein was normalized to that of β-actin. Data are expressed as Mean ± SD (n = 8). ^#^p < 0.05 vs ND group and *p < 0.05 vs HFD group.

The hepatic gene expressions of fatty acid synthase (FAS) and acetyl-CoA carboxylase (ACC), the enzymes involved in cholesterol and fatty acid synthesis, were significantly higher in HD group compared to ND group (Fig. 8A). However, the expressions of FAS and ACC genes were significantly decreased in HFD-fed animals when exposed to the higher and lower doses of mSJH, respectively. While the gene expressions of carnitine palmitoyl transferase 1α (CPT1α) and AMP-activated protein kinase α (AMPKα), the crucial enzymes in fatty acid metabolism (β-oxidation); the proteins related to cholesterol transport and uptake (LDLR, SR-B1, APOA, ABCA); and the enzymes participating in the rate-limiting steps of bile acid synthesis were lower in HFD group compared to ND group. The expressions of all of these genes in the liver of HFD-fed animals were increased in response to the treatment with both lower and higher doses of mSJH (except for the effect of lower dose on CYP7A1gene expression).

Our Western blotting results demonstrated higher expression level of 3-hydroxy-3-methyl-glutaryl-coenzyme A reductase (HMGCoAR) in the liver of HFD group compared to ND group (42% increase). However, treatment of HFD-fed rats with mSJH reduced the expression of this enzyme (33% and 35% decreases vs. ND group at lower and higher doses, respectively). While an exposure of HFD-fed animals to mSJH elevated the hepatic expression of CPT1α (551% and 480% increases vs. ND group at lower and higher doses, respectively). The hepatic level of phosphorylated-ACC (p-ACC) was lower in the liver of HFD group compared to ND group (67% decrease). However, the level of p-ACC in HFD-fed mice was increased in response to the treatment with both lower and higher doses of mSJH.

### Effect of betaine, a constituent of mSJH, on the lipid accumulation and expression of genes related to fatty acid metabolism

To further understand the underlying molecular mechanism(s) of action of mSJH against HS, the impact of this herbal formulation and its potent bioactive compound betaine on the lipid deposition and expression of genes that are involved in lipid metabolism was investigated using FFAs-induced HepG2 cells as a model. There was no adverse impact of betaine on the cell viability up to 60 μM of concentration (Fig. S6A). Treatment with 20, 40, and 60 μM of betaine significantly decreased the lipid accumulation in FFAs-treated cells as demonstrated by Oil Red O staining (Fig. S6B). Exposure of the FFAs-induced cells to both 20 and 60 μM of betaine markedly elevated the p-AMPK (271% and 576% increases, respectively) and CPT1α (659% and 956% increases, respectively) levels, quite comparable to the effects of 400 μM mSJH.

## Discussion

In the present study, the impact of mSJH on HS was examined through a series of *in vitro* and *vivo* experiments using human hepatocellular carcinoma cell line (HepG2) and HFD-fed mice models^23^,^24^,^25^. HepG2 cells were induced by a mixture of oleic and palmitic acids as these two fatty acids represent the most abundant components of hepatic triglyceride pool in both normal subjects and patients suffering from NAFLD^26^. In a previous study, treatment of HepG2 cells with a combination of oleic and palmitic acids at a certain ratio induced efficient intracellular lipid accumulation with the triglyceride content comparable to that found in human liver with steatosis^23^. In agreement with this, we found that exposure of HepG2 cells to a mixture of oleic and palmitic acids (FFAs) at a ratio of 2:1 (w/w) for 24 h resulted in significantly increased intracellular fat accumulation (114% augmentation vs untreated). Concomitantly, intracellular TC and TG levels were also significantly augmented in FFAs-treated cells compared to untreated. Furthermore, an earlier study reported the pathophysiological and metabolomic alterations associated with NAFLD development in a C57BL/6J mouse model in which NAFLD was induced by feeding a HFD for 4, 8, 12, and 16 weeks^24^. Besides, mice fed a HFD showed similar metabolic features observed in human NASH with obesity, impaired glucose tolerance, insulin resistance, dyslipidemia and augmented expression of regulators of lipogenesis and proinflammatory cytokines^25^. In keeping with these, we found that the feeding c57BL/6J mice with HFD for 10 weeks resulted in significant increases in the liver weight, hepatic fat deposition, liver and serum contents of TG and TC, and oxidized hepatic lipid content, as well as the activities of hepatic GOT and GPT, the key characteristics of NAFLD. Further, histological analyses of liver suggested hepatic fatty degeneration in HFD group.

Obesity and dyslipidemia are among the factors playing a key role in the onset and development of NAFLD^27^. Emerging evidence based on *in vitro* and *in vivo* experiments support the anti-obesity and hypolipidemic activities of SJH or its ingredients^19^,^20^,^21^. In agreement with these, our *in vitro* results showed that the lipid accumulation and the content of TC and TG in FFAs-induced cells were significantly suppressed by mSJH treatment in a concentration-dependent manner. In parallel, our *in vivo* study demonstrated that the weight, lipid deposition, contents of TC and TG, and activities of GOT and GPT in the liver of HFD-fed mice were attenuated by mSJH treatment in a dose-dependent manner. The histological analyses of liver tissues further revealed that mSJH prevented hepatic degenerative changes induced by feeding HFD. Moreover, treatment of HFD-fed mice with both lower and higher doses of mSJH significantly increased the fecal bile acid content. Bile acid in normally-functioned-liver acts as a primary pathway for cholesterol catabolism^28^ and major cholesterol-scavenging mechanism^29^,^30^. A previous study showed an increase in the bile acid excretion in the feces of HFD-fed mice in response to the treatment with oat β-glucan, an anti-obesity agent, indicating that there was binding of bile acids by the oat β-glucan^31^. Based on the earlier reports indicating that hyperlipidemia is closely related to the onset and development of hepatic steatosis^3^,^32^ and our current findings, it is conceivable that mSJH may have a protective effect against HS.

Previous *in vivo* and *in vitro* studies have shown that the hypolipidemic effect of herbal extracts can play a key role in preventing lipid-induced NASH^33^. Oxidative stress is also known to be a major contributing factor to the pathogenesis of NAFLD. Exposure of mitochondria to the excessive intracellular milieu of lipids facilitates the reduction of β-oxidation and accelerates the production of reactive oxygen species (ROS)^26^,^34^. ROS provokes lipid peroxidation followed by induction of the inflammatory response as well as activation of hepatic stellate cells, ultimately leading to fibrogenesis^35^,^36^. In keeping with a previous report^18^, in our study, mSJH exhibited marked antioxidant properties in terms of its *in vitro* DPPH radical and ROS scavenging activities and ability to suppress the hepatic lipid oxidation in HFD-fed mice. Earlier *in vivo* and *in vitro* studies demonstrated that antioxidant properties of herbal extracts may play a significant role in protecting against lipid-induced NASH^37^. In particular, antioxidants are known to reduce ER stress^38^ which is a characteristic feature of liver and adipose tissues of humans suffering from NAFLD and/or obesity^39^. Based on the above information, it is conceivable that because of its appreciable antioxidant activities, mSJH can combat fatty acids-induced ER stress in cells. Such an event has also been reflected in a study reporting that the extract of *Morus alba* leaf, the major constituent of SJH, attenuates ER stress and this effect is principally driven by its antioxidant activity^40^.

Next we performed the analysis of expression of vital genes involved in lipoprotein signaling and cholesterol metabolism both *in vitro* and *in vivo* using PCR microarray and qRT-PCR, respectively. Heat map combined with the hierarchical clustering analysis of microarray data from cell-based studies showed that the clustering of these genes in the FFAs + mSJH-treated group was most relevant to that in the FFAs-induced group but farthest from that in the normal group. This signifies that the FFAs-treated and FFAs + mSJH-treated groups are closely related in terms of the gene expression profile. Therefore, it is conceivable that mSJH treatment has an effect on a limited number of genes in a FFAs-induced steatosis model. Indeed, in our analysis, among the 89 genes analyzed, only 14 genes showed higher (≧2 times) expression and 3 genes showed lower (≦0.5 times) expression in the FFAs + mSJH-treated group compared with the FFAs-induced group. Among the upregulated genes, 8 genes exhibited ≧2 times higher expression vs. FFAs + pravastatin group. Especially, the expression level of the leptin gene in FFAs + mSJH-treated group showed marked differences, compared to the FFAs-treated group (3 fold increase) and FFA + pravastatin group (19 fold increase). Based on these findings, it is conceivable that mSJH modulates the lipid metabolism genes differentially compared to pravastatin.

Our further *in vivo* studies using real-time PCR revealed the influence of mSJH on the lipid metabolism genes in the liver of HFD-fed mice, especially on the expression of genes related to cholesterol and fatty acid synthesis (decrease in FAS and ACC), fatty acid metabolism (increase in CPT1α and AMPKα), cholesterol transport (increase in LDLR, SR-B1, APOA, ABCA), and bile acid synthesis (increase in CYP7A1, CYP8B1, and CYP27A1). Activation of lipogenic genes including FAS and ACC are highly related to elevated triglycerides level; thus inhibition of these proteins is a potential therapeutic strategy for dyslipidemia^41^,^42^. Agonists for groups of genes related to β-oxidation of fatty acid including AMPKα and CPT1α are also regarded as hypolipidemic agents^43^. It has been found that CPT1 plays a protective role against fatty acid-induced adipocyte dysfunction suggesting that pharmacological activation of CPT1 might represent a promising strategy for the prevention and treatment of obesity related metabolic diseases^44^. In keeping with the gene expression data, our western blotting analyses also revealed that exposure of HFD-fed mice to mSJH increased the hepatic level of CPT1α protein. Furthermore, mSJH treatment, especially at lower dose induced the phosphorylation of hepatic ACC in HFD-fed mice, thereby causing inactivation of this enzyme^45^. Our western blotting analyses also exhibited that treatment of HFD-fed mice with mSJH markedly reduced the hepatic level of HMG-CoA reductase, a principal enzyme in cholesterol biosynthesis pathway and the main target of statins^46^.

Reverse cholesterol transportation is a major factor in regulating and restoring lipid profiles in dyslipidemia. It has been found that upregulated gene expression of LDL receptor, SR-B1 and ABCA removes excessive cholesterol^47^,^48^. The current line of evidence indicates a significant role of the CYP family of proteins in lipid metabolism. By catalysis of the initial steps in various pathways of cholesterol degradation, CYP450 7A1, 27A1, 11A1, and 46A1 were found to play vital roles in the regulation of cholesterol homeostasis^49^. As a rate-limiting enzyme for production of bile acid, CYP7A1, CYP8B1 and CYP27A1 are core hepatic enzymes to be examined^28^. Among these enzymes, CYP7A1 initiates and regulates the rate-limiting step of cholesterol degradation and bile acid biosynthesis in the liver by converting cholesterol to 7α-hydroxycholesterol^50^. Our results on PCR array revealed that an exposure of FFAs-induced cells to mSJH increased the expression of CYP7A1 gene by 18%. This is also in keeping with our *in vivo* studies as stated above where treatment of HFD-fed mice with high dose of mSJH increased the gene expression of CYP7A1, CYP8B1, CYP27A1, significantly for the last two proteins. Based on these findings and the potential of CYP family for decreased lipid accumulation in animal models^51^, it is conceivable that modulation of CYP family by mSJH might contribute to the beneficial effects of this herbal formulation observed in our study. More specifically, mSJH treatment may promote reverse cholesterol transport and eventually increased excretion of cholesterol in the form of bile acids via feces.

Our real-time PCR and western blotting analyses confirmed significantly elevated gene and protein expression levels of leptin in FFAs + mSJH-treated cells compared to FFAs-alone treated cells, in alignment with our findings in microarray study, suggesting that the effect of mSJH may be related to the leptin signaling pathway. Some emerging evidence, including that of Jeong *et al*.^52^, has indicated that the regulation of leptin expression is one of the major factors in the prevention of obesity and associated lipid accumulation in the liver by Gyeongshingangjeehwan, a traditional Korean medicine^52^. Although leptin is synthesized primarily in the adipocyte tissues, a small quantity of this protein is also secreted from the hepatic tissues^53^, carcinoma cell line (HepG2)^54^, and stellate cells of liver during its progression to fibrosis and/or cirrhosis stages^55^. Lack of hepatic leptin signaling results in increased lipid accumulation in the liver enriched with larger and more triglyceride-rich VLDL particles, suggesting a vital role of hepatic leptin signaling in the regulation of triglyceride metabolism^15^.

Activation of AMPK or ACC is subordinated by leptin signaling^9^. More specifically, leptin signaling activates AMPK through phosphorylation^56^, thereby modulating a downstream signaling cascade that results in the inhibition of phosphorylation of SREBP1c^57^, leading to the suppression of lipogenesis. In our study, a marked increase in the phosphorylation of AMPK along with the decreased intracellular lipid accumulation were observed in FFAs-treated cells in response to the exposure to mSJH, suggesting that the anti-lipogenic action of this herbal formulation is mediated through leptin-cell interaction. Moreover, treatment of the HFD-fed mice with both lower and higher doses of mSJH also significantly augmented the expression of hepatic AMPK-α gene. Induced AMPK is known to trigger phosphorylation and inhibition of ACC and thereby, prevents liponeogenesis^58^. Indeed, in our *in vitro* study a significant increase in the phosphorylation of ACC was observed in FFAs-treated cells in response to co-exposure to mSJH at 400 μg/ml concentration, in alignment with our *in vivo* findings as stated above. Inactivation of ACC by AMPK also facilitates fatty acid utilization, leading to fat burning in liver and muscle^58^. On the other hand, emerging evidence indicates the role of PPAR-γ in the onset and development of HS^59^. In mice, high fat diet-induced HS was found to be associated with elevated expression of PPAR-γ^60^. In another study, hepatic overexpression of PPAR-γ 1 in PPAR-α knockout (PPAR-α-KO) mice was shown to induce adipocyte-specific gene expression patterns in the livers of the animals^61^. Based on these findings, it was proposed that excess PPAR-γ activity can lead to the development of adipogenic hepatic steatosis^61^. In addition, liver-specific disruption of PPAR-γ in genetically obese mice was shown to prevent HS^62^, suggesting that a decrease in hepatic PPAR-γ pool is therapeutically beneficial for HS. In our study, a significant decrease in PPAR-γ level was observed in FFAs-induced cells when co-treated with mSJH at 400 μg/ml, suggesting that downregulation of PPAR-γ may contribute to the protective effect of this herbal medicine against HS.

Leptin is also known to stimulate lipid oxidation, ultimately leading to a decrease in the intracellular level of TG^63^. In hepatic tissue, fatty acid oxidation is primarily regulated by the level of CPT1^64^, which is mainly represented by CPT1α (the liver isoform). Using bovine aortic endothelial cells as a model, leptin was shown to induce CPT1α activity^65^. In our *in vitro* study, the protein expression of CPT1α was markedly elevated in FFAs-treated cells upon co-exposure to mSJH at 400 μg/ml concentration, in agreement with our *in vivo* findings as stated above, suggesting that this effect may be mediated through leptin. Accumulating evidence also indicates that like PPAR-γ, C/EBPα plays a vital role in hepatic lipid metabolism. Knockout of C/EBPα in ob/ob mice significantly reduced the TG level and attenuated the expression of lipogenic genes^66^. In addition, using mice fed a high-fat diet as a model, downregulation of expression of lipogenic genes including C/EBPα in the liver was shown to play an important role in attenuating HS^67^. In our study, the protein expression of C/EBPα was significantly reduced in FFAs-treated cells following co-exposure to mSJH at 200 and 400 μg/ml concentrations, further explaining the molecular basis of action of this formulation against liponeogenesis and HS.

A previous report has shown a rapid inhibition of leptin signaling by glucocorticoids both *in vitro* and *in vivo*^68^. In our study, we found that an exposure to dexamethasone not only crippled leptin signaling, but also decreased the leptin protein level in FFAs-treated cells. Furthermore, mSJH-induced expression of leptin as well as mSJH-triggered phosphorylation of AMPK in FFAs-treated cells were markedly inhibited upon co-treatment with dexamethasone. Taken all, it is conceivable that the HepG2 cells used as a model in our study can interact with self-secreted leptin and can be modulated through leptin signaling. Probably, mSJH acts through leptin signaling pathway to combat FFAs-induced hepatic steatosis.

The UPLC fingerprinting of mSJH demonstrated the appearance of peaks that tentatively represent potential active compounds derived from its principal ingredients including cyanidin-3-*O*-rutinoside, betaine, kukoamine A, atractylodin, and atractylenolide II. Among them, betaine is well known for its inhibitory effect on the onset and development of fatty liver^69^. To further confirm the bioactive properties of betaine, we examined the effect of this compound on the hepatic steatosis using FFAs-induced HepG2 *in vitro* cell model as mentioned above. Our results revealed that an exposure of FFAs-treated cells to mSJH at 400 μg/ml and 60 μM concentrations of betaine, which were non-toxic, significantly ameliorated lipid accumulation. Moreover, the western blotting data demonstrated increase in the levels of both CPT1α and phosphorylated AMPKα in FFAs-treated cells by 20 and 60 μM of betaine. As a modulator of energy metabolism, activation of AMPK by phosphorylation has therapeutic effects on fatty liver, insulin resistance, and hyperlipidemia^70^,^71^,^72^. It has been reported that betaine elevates level of phosphorylated AMPK and protects against non-alcoholic fatty liver (NAFL) *in vivo*^73^.

In conclusion, the present study reveals that mSJH has an attenuating effect on hepatic steatosis both *in vitro* and *vivo* models. This beneficial effect of mSJH is likely to be mediated through increased expression of leptin that may trigger signaling cascades involved in lipid metabolism pathways. Our study also suggests that betaine significantly contributed to the beneficial effects of mSJH on leptin signaling.

## Methods

### Herbal extraction and formulation preparation

Dried forms of Mori Fructus [Sangsimja in Traditional Korean Medicine (TKM)], Atractylodis Rhizoma (Changchul in TKM), and Lycii Radicis Cortex (Jigolpi in TKM) were purchased from the Department of Medicine, Dongguk University International Hospital (Goyang, Republic of Korea). Extraction and preparation of the herbal formulation were performed according to our laboratory-optimized procedure with modifications. Briefly, these three herbs were ground separately into powder form which was then combined together (3:1:1, w/w, respectively). The resultant mixture was subjected to reflux extraction with 10 vol. of 30% ethanol for 1 h, followed by evaporation at 95 °C using a rotary evaporator system (Buchi, Flawil, Switzerland) The extract was filtered through a 0.2 μm syringe filter (Merck Millipore, Billerica, MA, USA) and concentrated by vacuum evaporation at 60 °C. The final product was stored at −20 °C prior to experimental use.

### Cell Culture and Treatments

HepG2 cells (#88065, Korea Cell Line Bank, Seoul, Republic of Korea) were cultured in Dulbecco’s Modified Eagle Medium (DMEM, Gibco, Carlsbad, CA, USA) supplemented with 10% fetal bovine serum (Gibco), and 100 U/mL penicillin and streptomycin (Gibco). Cells were grown in an incubator at 37 °C under a humidified environment of air containing 5% CO_2_. Cells were maintained consistently to approximately 70–80% confluence. For experimental purposes, cells were seeded at a density of 5 × 10^5^ or 1 × 10^5^ cells per well in 6- or 24-well plates, respectively. After 24 h of culturing, the medium was removed and replaced with fresh DMEM containing 1% BSA (Amresco, Cleveland, OH, USA). Hepatic steatosis-mimetic conditions were induced by treating the cells in 6- or 24-well plates for 24 h with a mixture of free fatty acids [oleic acid: palmitic acid, (Sigma-Aldrich, St. Louis, MO, USA) 2:1, w/w] dissolved in the culture media (the final lipid concentration was kept at 2 mM)^74^. Following this, the cells were washed with Dulbecco’s phosphate-buffered saline (DPBS, Gibco), pH 7.4 and then fresh DMEM containing DPBS (normal) or different concentrations of mSJH, wherever applicable was added. The cells were incubated in this condition for 24 h (Western blot) or 48 h (ORO staining)prior to experimental use. For the evaluation of cytotoxicity of mSJH alone, the cells were processed identically as mentioned above except that they were not induced by FFAs.

### Assessment of the *in vitro* hepatic steatosis model

#### Determination of intracellular lipid accumulation by Oil Red O staining

After the completion of desired treatment schedule, HepG2 cells were washed with DPBS and then fixed with 10% formalin solution for 5 min at room temperature followed by gentle washing with 60% isopropanol and then stained with the working solution of Oil Red O (Sigma-Aldrich) in 60% isopropanol for 15 min. The stained hepatocytes were washed with distilled water several times for removal of unincorporated dye. Cells were examined under an inverted microscope (DMI 6000, Leica, Jena, Germany) and the images were captured. The intracellular stains were re-dissolved in 100% isopropanol for 10 min, and the resultant solutions were transferred to a 96-well plate for measurement of absorbance at 520 nm using a microplate reader (VersaMax, Molecular Devices, CA, USA).

#### Measurement of intracellular triglyceride and total cholesterol

Following the desired treatments, cells were washed with DPBS, scraped and transferred into tubes and then centrifuged at 3000 rpm for 5 min. The resultant cell pellets were washed and dissolved in radioimmunoprecipitation assay (RIPA) buffer (Thermo Fisher scientific, Waltham, MA, USA). The amounts of TG and TC were measured using a Triglyceride and total cholesterol assay kit (Asan pharmacology, Seoul, Republic of Korea) and normalized with the cellular content of protein as determined using a BCA protein assay kit (Thermo Fisher Scientific).

### Animals

Thirty two, 4-weeks old C57BL/6 J mice were purchased from Koatech (Pyeongtaek, Gyeonggi-do, Republic of Korea). Mice were randomly distributed into eight cages (four animals per cage) and housed under 12 h light/dark cycle, constant temperature (25 °C) and humidity (50–60%). They were provided with standard AIN93G rodent diet and access to water *ad libitum* and were acclimatized in this condition for one week. The animals were then divided randomly into four groups. Normal diet group: fed AIN93G diet (ND, n = 8); high-fat diet group (HFD, n = 8), high-fat diet + lower dose mSJH group (LD, n = 8), and high fat diet + higher dose mSJH group (HD, n = 8): fed 60% high-fat diet with or without the specified mSJH treatments for 10 weeks. All diets were purchased from Saeronbio (Uiwang, Gyeonggi-do, Republic of Korea). The mSJH was dissolved in distilled water and administered orally using gavage at a dose of 100 mg/kg/day in case of LD or 600 mg/kg/day in case of HD groups for 10 weeks. Same volume of distilled water without mSJH was administered to the animals of ND and HFD groups. Following the completion of experimental schedule, all mice were starved for 12 h and then sacrificed under anesthesia induced with zoletil (Virbac, Fort Worth, TX, USA). Liver, fat tissues, and kidney were excised immediately, washed in ice-cold PBS, pH 7.4, dried, weighed and stored at − 80 C° until used. A portion of the liver tissue was preserved in 10% neutral buffered formaldehyde for further histological analyses. Small quantities of tissues were also kept separately in Invitrogen™ RNA*later*™ stabilization solution (Thermo Fisher Scientific) and then stored at − 80 °C for future RNA isolation. Following the collection of blood samples, sera were separated by centrifugation at 3000 rpm for 20 min. The fecal samples were collected weekly in individual tubes and stored at − 80 C° until analyzed. The animal experiment was performed according to the international guidelines (Guide for the Care and Use of Laboratory Animals, Institute of Laboratory Animal Resources, Commission on Life Sciences, National Research Council, USA; National Academy Press: Washington D.C., 1996). The design and protocols for the animal experiment were approved by the Institutional Animal Ethical Committee of Dongguk University (No. 201411115) prior to the study.

### Histology

For histological analyses, portions of the formaldehyde-preserved liver tissues were embedded in FSC 22 ^®^ frozen section media (Leica, Richmond, IL, USA) at −15 °C for Oil Red O (ORO) staining and at −25 °C for hematoxylin & eosin (H&E) staining. Tissues were then sectioned at 5 μm thickness using a CM1860 cryostat (Leica Biosystems, Nussloch GmbH, Germany). Sections were fixed in 10% formalin solution and then stained with ORO (Sigma-Aldrich) for 30 min or with H&E stain (Sigma-Aldrich) for 3 min. The ORO-stained sections were counterstained with hematoxylin for three dips. Finally, the stained sections were observed at 200X magnification under a Leica DMI 6000 B fully automated inverted microscope equipped with a Leica DFC480 camera (Leica Microsystems GmbH, Wetzlar, Germany).

### Serum analyses

Serum HDL, triglycerides (TG), and total cholesterol (TC) levels were measured using commercial colorimetric kits (Asan pharmacology) following the kit manufacturer’s instructions. Serum hepatic enzyme activity was determined using glutamic oxaloacetic transaminase/glutamic pyruvic transaminase (GOT/GPT) test kit (Asan pharmacology) as per the kit manufacturer’s protocol.

### Analyses of hepatic lipid (TG, TC) contents and lipid peroxidation (MDA)

After washing with ice-cold DPBS, the liver tissues were homogenized in DPBS on ice using an Vibra-Cell™ ultrasonic liquid processor (Sonics & Materials, Newtown, CT, USA). The tissue homogenate was centrifuged at 12000 rpm for 5 min at 4 °C and the resultant supernatant was separated and used as the sample. The TG and TC contents of the samples were measured using commercial kits (Asan pharmacology). For the determination MDA as an indicator of lipid peroxidation, the sample was mixed with 2-thiobarbituric acid (TBA) (Sigma) at a final concentration of 8.1% (w/v). The mixture was incubated at 95 °C for 1 h to allow the formation of red-colored TBA-chromogen. The reaction was then stopped by cooling, following which 1 ml of distilled water was added. Subsequently, 5 ml of a mixture of pyridine and 1-butanol (Sigma-Aldrich) (1: 15, v/v) was added to the reactants, vortexed thoroughly and then centrifuged at 3000 rpm for 30 min. The supernatant was transferred to a multi-well plate and absorbance was read at 532 nm on a microplate reader (VersaMax, Molecular Devices). The same volume of distilled water was used as blank and 1,1,3,3-tetraethoxy propane (Sigma-Aldrich) at various concentrations served as the standard.

### Determination of fecal lipid content

Fecal lipid content was measured following the Folch’s method^75^ with slight modification. Briefly, after collection, fecal samples (100–200 mg) were freeze-dried for 48 h. After weighing, 1 ml of distilled water was added to the dried feces, mixed thoroughly and then homogenized. To the homogenate, 4 ml of chloroform and 2 ml of methanol were added and mixed thoroughly. The mixture was incubated at RT for 48 h. The bottom fraction containing the fecal lipids was isolated, completely dried and eventually suspended in 2-isopropanol. The supernatant portion of the suspension was subjected to TG and TC analyses using commercial kits (Asan pharmacology).

### Analysis of fecal bile acid content

Dried feces in powder form were mixed with sodium borohydride solution at a final concentration of 2 mg/ml. To this, 2 N HCl and 10 N NaOH were added for saponification of the bile acid. After heating at 120 °C for 12 h, samples were filtered and dried completely and eventually eluted with pure water. Eluted bile acid was applied to Bond Elut C18 cartridges (Agilent Technologies, Santa Clara, CA, USA) pre-washed with 100% methanol. The cartridges were washed with 20% methanol and finally the samples were eluted with 100% methanol. Eluted bile acid was extracted by drying at 60 °C for 48 h and subsequently re-eluted. The concentration of the bile acid was determined using a commercial bile acid kit (Crystal Chem, Downers Grove, IL, USA) according to the instructions provided by the manufacturer of the kit.

### Oral glucose tolerance test (OGTT)

One week prior to sacrifice, the mice were subjected to oral glucose tolerance test. The OGTT was conducted on the animals fasted overnight (12 h) by oral administration of glucose (prepared in autoclaved distilled water) at a dose of 1.5 g/Kg body weight. Blood collected from the tail vein was used as the sample. Blood glucose levels were measured at five different time points (0, 30, 60, 90, and 120 min) using a glucose meter (ACCU-CHEK, Roche Applied Science, Basel, Switzerland).

### Quantitative real-time PCR

Following desired treatments, total RNA was extracted from HepG2 cells using TRIZOL reagent (Invitrogen, Carlsbad, CA, USA) according to the reagent manufacturer’s instructions. While, mice liver tissues stored in RNA*later*™ stabilization solution was shortly homogenized followed by extraction of total RNA using TRIZOL reagent (Invitrogen). The RNA samples were quantified and their integrity was determined by measuring of ODs at 260 and 280 nm. The extracted RNA was transcribed using an AccuPower^®^ RT PreMix kit (Bioneer, Daejeon, Republic of Korea) and oligo dt primers (Invitrogen) according to the kit manufacturer’s protocol in a final volume of 20 μl. The real-time PCR amplification reaction was performed using a LightCycler instrument and LightCycler^®^ FastStart DNA Master SYBR Green kit (Roche Applied Science, Indianapolis, ID, USA), and the listed primers (Table S1). The reaction was performed following the product manual of the kit manufacturer in a total reaction volume of 20 μl containing PCR mix, 1 μg of cDNA, and gene-specific primers (10 pmol for each). The relative gene expression of the selected proteins was calculated as 2^−ΔCt^ using β-actin as a housekeeping gene for normalization, where C_t_ represents the crossing threshold value and ΔC_t_ = C_t (leptin gene)_ − C_t (β-actin gene)_.

### Gene expression profiling by PCR array

After desired treatments of HepG2 cells, total cellular RNA was extracted and reverse-transcribed using a similar method as described above for quantitative real-time PCR. Expression of genes involved in lipid metabolism and cholesterol was studied by PCR array using a 96-well Human Lipoprotein Signaling & Cholesterol Metabolism RT^2^Profiler PCR Array Kit (Qiagen, Hilden, Germany) and LightCycler 480 PCR system (Roche, Basel, Switzerland) according to the kit manufacturer’s protocol.

### Western blotting

HepG2 cells were washed with ice-cold DPBS and lysed using RIPA buffer containing protease and phosphatase inhibitor cocktail (Roche Diagnostics, Basel, Switzerland) on ice for 30 min. While after washing with ice-cold DPBS, mice liver tissues were homogenized in DPBS containing protease and phosphatase inhibitor cocktail (Roche Diagnostics) on ice using an Vibra-Cell™ ultrasonic liquid processor (Sonics & Materials). The cell lysates and tissue homogenates were centrifuged at 14,000 rpm for 30 min at 4 °C to remove the insoluble materials and the resultant supernatants were stored at −80 °C until further use. The protein content of the supernatants was determined using a BCA protein assay kit (Thermo Fisher Scientific). The samples were subjected to denaturation at 100 °C in Laemmli sample buffer (BioRad, Hercules, CA, USA) containing 5% β-mercaptoethanol. Forty microgram of protein was resolved in SDS-PAGE gel and transferred to a PVDF membrane (GE Healthcare UK Ltd., Buckinghamshire, UK) using a Mini Trans-Blot^®^ Electrophoretic Transfer Cell device (BioRad). Membranes were blocked with Tris-buffered saline (TBS) containing 0.1% Tween 20 (TBST) and 5% non-fat dried milk (BD Falcon, Sparks, MD, USA) for 60 min and then incubated overnight with anti-phospho-AMPK (Thr 172), anti-phospho ACC (Ser 79), anti-SREBP1c, anti-CPT1α, anti-PPAR-γ, anti-C/EBPα (Cell Signaling Technology, Danvers, MA, USA), anti-leptin (Santa cruez, Dallas, TX, USA), and anti-HMG-CoAR (Abcam, Cambridge, UK) antibodies (1:1000 dilution in TBST containing 3% skim milk or BSA) at 4 °C. After thorough washing with TBST, the membranes were incubated for 90 min with the appropriate horseradish peroxidase-conjugated anti-IgG secondary antibodies (Cell Signaling Technology) (1:2000 dilution in TBST containing 1% skim milk) and the immunoreactive bands were detected on a Las3000 imaging system (Fujifilm, Tokyo, Japan) using Supex ECL reagent (Neuronex, Seoul, Republic of Korea). The membranes were then stripped in a buffer [62.5 mM Tris–HCl (pH 6.7) containing 2% SDS and 100 mM β-mercaptoethanol] and reprobed with anti-AMPK and anti-ACC antibodies, wherever applicable and processed identically as mentioned above. Finally, all the membranes were reprobed with β-actin in a similar way using an anti-β-actin antibody (Cell Signaling Technology).

### Studies on dexamethasone-mediated inhibition of leptin signaling

We opted dexamethasone as a leptin signaling inhibitor^68^ in order to understand the probable underlying molecular mechanism(s) of mSJH-induced action of leptin against hepatic steatosis. After 24 h of treatment with 2 mM FFAs to induce HS as stated above, HepG2 cells were washed and fresh media was added. Dexamethasone (Sigma-Aldrich) dissolved in sterile DPBS was added to the media at a final concentration of 100 μM at different intervals (0, 1, 3, 6, 12, and 24 h) to examine the impact of this compound on the time-dependent expression of leptin in FFAs-treated cells. In other experiments, the FFAs-induced cells were exposed to 100 nM dexamethasone for 30 min followed by the treatment with mSJH at 0, 200, 400 μg/ml concentrations for 12 h (Western blot) or 24 h (ORO staining). The cells were then subjected to Western blotting or Oil Red O staining.

### Statistical analysis

The data are expressed as mean ± standard deviation (SD) from at least three independent determinations. All data were subjected to statistical analysis using the Statistical Package for Social Science (SPSS) software program (version 10.0; SPSS, Chicago, IL, USA). A one-way ANOVA, followed by Bonferroni’s post hoc analysis, was carried out to determine the statistical significance. The differences were considered statistically significant at p < 0.05. Correlation heatmap analysis and interpretation of data derived from Lipoprotein Signaling & Cholesterol Metabolism PCR array were performed using RT^2^ Profiler PCR Array Data Analysis software version 3.5 (Qiagen).

## Additional Information

**How to cite this article**: Lim, D.-W. *et al*. Modified SJH alleviates FFAs-induced hepatic steatosis through leptin signaling pathways. *Sci. Rep.* **7**, 45425; doi: 10.1038/srep45425 (2017).

**Publisher's note:** Springer Nature remains neutral with regard to jurisdictional claims in published maps and institutional affiliations.

## Supplementary Material

Supplementary Data

## References

[b1] Milanski M. D. . Saturated fatty acids produce an inflammatory response predominantly through the activation of TLR4 signaling in hypothalamus: implications for the pathogenesis of obesity. J Neurosci 29, 359–370 (2009). 19144836 10.1523/JNEUROSCI.2760-08.2009 PMC6664935

[b2] Jo H. C. . Endoplasmic reticulum stress induces hepatic steatosis via increased expression of the hepatic very low-density lipoprotein receptor. Hepatology 57, 1366–1377 (2013). 23152128 10.1002/hep.26126

[b3] Sass D. A., Chang P. & Chopra K. B. Nonalcoholic fatty liver disease: a clinical review. Digest Dis Sci 50, 171–180 (2005). 15712657 10.1007/s10620-005-1267-z

[b4] Akabame S. H. . Evaluation of vulnerable coronary plaques and non-alcoholic fatty liver disease (NAFLD) by 64-detector multislice computed tomography (MSCT). Circulation J 72, 618–625 (2008). 10.1253/circj.72.618 18362435

[b5] Chalasani N. Y. . The diagnosis and management of non-alcoholic fatty liver disease: practice guideline by the American Gastroenterological Association, American Association for the Study of Liver Diseases, and American College of Gastroenterology, American Gastroenterological Association. Gastroenterology 142, 1592–1609 (2012). 22656328 10.1053/j.gastro.2012.04.001

[b6] Farrell G. C. & Larter C. Z. Nonalcoholic fatty liver disease: from steatosis to cirrhosis. Hepatology 43, S99–S112 (2006). 16447287 10.1002/hep.20973

[b7] Williams K. H. . Diabetes and nonalcoholic Fatty liver disease: a pathogenic duo. Endocr Rev 34, 84–129 (2013). 23238855 10.1210/er.2012-1009

[b8] Fasshauer M. & BLuher M. Adipokines in health and disease. Trends Pharmacol Sci 36, 461–470 (2015). 26022934 10.1016/j.tips.2015.04.014

[b9] Deng Y. & Scherer P. E. Adipokines as novel biomarkers and regulators of the metabolic syndrome. Ann N Y Acad Sci 1212, E1–E19 (2010). 10.1111/j.1749-6632.2010.05875.x PMC3075414 21276002

[b10] Abella V. . Adipokines, metabolic syndrome and rheumatic diseases. J Immunol Res 2014, 343746 (2014). 24741591 10.1155/2014/343746 PMC3987880

[b11] Minokoshi Y., Toda C. & Okamoto S. Regulatory role of leptin in glucose and lipid metabolism in skeletal muscle. Indian J Endocrinol Metab 16, S562–568 (2012). 23565491 10.4103/2230-8210.105573 PMC3602985

[b12] Patel S. . Leptin: linking obesity, the metabolic syndrome, and cardiovascular disease. Curr Hypertens Rep 10, 131–137 (2008). 18474180 10.1007/s11906-008-0025-y

[b13] Ferrante M. C. . Polychlorinated biphenyls (PCB 101, PCB 153 and PCB 180) alter leptin signaling and lipid metabolism in differentiated 3T3-L1 adipocytes. Toxicol Appl Pharm 279, 401–408 (2014). 10.1016/j.taap.2014.06.016 24978599

[b14] Ho M. . Leptin-mediated inhibition of the insulin-stimulated increase in fatty acid uptake in differentiated 3T3-L1 adipocytes. Metabolism 55, 8–12 (2006). 16324913 10.1016/j.metabol.2005.06.013

[b15] Huynh F. K. . A role for hepatic leptin signaling in lipid metabolism via altered very low density lipoprotein composition and liver lipase activity in mice. Hepatology 57, 543–554 (2013). 22941940 10.1002/hep.26043

[b16] Lee D. J. Peroxynitrite Scavenging Activity of Samjunghwan. Korean J Orient Int Med 27, 178–187 (2006).

[b17] Kim H. G. . Evaluation of Samjunghwan, a traditional medicine, for neuroprotection against damage by amyloid-beta in rat cortical neurons. J of ethnopharmacology 130, 625–630 (2010). 20538052 10.1016/j.jep.2010.05.040

[b18] Han K. S. . Anti-Oxidative and Anti-Obesity Effect of Combined Extract and Individual Extract of Samjunghwan. J Korean Med Obes Res 14, 47–54 (2014).

[b19] Song M. Y., Shambhunath B. & Kim H. J. Effect of Probiotics-Fermented Samjunghwan on Differentiation in 3T3-L1 Preadipocytes. J Korean Soc Food Sci Nutr 42, 1–7 (2013).

[b20] Kim H. G. . Study of Mori Fructus and Dried Mori Fructus Extracts on the Antioxidant Effect and the Inhibitory Effect on Adipocyte Differentiation. Korean Med Rehab 24, 1–13 (2014).

[b21] Peng C. H. . Mulberry water extracts possess an anti-obesity effect and ability to inhibit hepatic lipogenesis and promote lipolysis. J Agric Food Chem 59, 2663–2671 (2011). 21361295 10.1021/jf1043508

[b22] Liu L. K. . Effects of mulberry (Morus alba L.) extracts on lipid homeostasis in vitro and *in vivo*. J Agric Food Chem 57, 7605–7611 (2009). 19630385 10.1021/jf9014697

[b23] Gomez-Lechon M. J. . A human hepatocellular *in vitro* model to investigate steatosis. Chem Biol Interact 165, 106–116 (2007). 17188672 10.1016/j.cbi.2006.11.004

[b24] Lai Y. S. . Mass-Spectrometry-Based Serum Metabolomics of a C57BL/6J Mouse Model of High-Fat-Diet-Induced Non-alcoholic Fatty Liver Disease Development. J Agric Food Chem 63, 7873–7884 (2015). 26262841 10.1021/acs.jafc.5b02830

[b25] Asgharpour A., Vincent R. & Min H. Of Mice and Men: A Review of Dietary Murine Models of Nonalcoholic Fatty Liver Disease (NAFLD) and How It Correlates to Human Disease. Austin J Clin Med 1, 5 (2014).

[b26] Yang S. Z. . Mitochondrial adaptations to obesity-related oxidant stress. Arch Biochem Biophys 378, 259–268 (2000). 10860543 10.1006/abbi.2000.1829

[b27] Angulo P. GI epidemiology: nonalcoholic fatty liver disease. Aliment Pharm Ther 25, 883–889 (2007). 10.1111/j.1365-2036.2007.03246.x 17402991

[b28] Li T. & Chiang J. Y. Regulation of bile acid and cholesterol metabolism by PPARs. PPAR Res 2009, 501739 (2009). 19636418 10.1155/2009/501739 PMC2712638

[b29] Charach G. . The role of bile Acid excretion in atherosclerotic coronary artery disease. Int J Vasc Med 2012, 949672 (2012). 21918722 10.1155/2012/949672 PMC3171758

[b30] St-Pierre M. V., Kullak-Ublick G. A., Hagenbuch B. & Meier P. J. Transport of bile acids in hepatic and non-hepatic tissues. J Exp Biol 204, 1673–1686 (2001). 11316487 10.1242/jeb.204.10.1673

[b31] Ji-Lin D., Ying-ying Z., Lin L., Rui-ling S. & Hong L. Effect of Oat Soluble and Insoluble beta-glucan on Lipid Metabolism and Intestinal Lactobacillus in High-fat Diet-induced Obese Mice. J Food Nutr Res 2, 510–516 (2014).

[b32] Abd El-Kader S. & El-Den Ashmawy E. M. Non-alcoholic fatty liver disease: The diagnosis and management. World J Hepatol 7, 846–858 (2015). 25937862 10.4254/wjh.v7.i6.846 PMC4411527

[b33] Jadeja R., Devkar R. V. & Nammi S. Herbal medicines for the treatment of nonalcoholic steatohepatitis: current scenario and future prospects. Evid-Based Compl Alt 2014 (2014). 10.1155/2014/648308 PMC4060323 24987431

[b34] Day C. P. & James O. F. Hepatic steatosis: innocent bystander or guilty party? Hepatology 27, 1463–1466 (1998). 9620314 10.1002/hep.510270601

[b35] Curzio M., Esterbauer H. & Dianzani M. U. Chemotactic activity of hydroxyalkenals on rat neutrophils. Int J Tissue React 7, 137–142 (1984). 3839769

[b36] Lee K. S., Buck M., Houglum K. & Chojkier M. Activation of hepatic stellate cells by TGF alpha and collagen type I is mediated by oxidative stress through c-myb expression. J Clin Invest 96, 2461 (1995). 7593635 10.1172/JCI118304 PMC185899

[b37] Jadeja R. D., Ranjitsinh V. & Nammi Srinivas. Herbal medicines for the treatment of nonalcoholic steatohepatitis: current scenario and future prospects. Evid-Based Compl Alt 2014 (2014). 10.1155/2014/648308 PMC4060323 24987431

[b38] Malhotra J. D. . Antioxidants reduce endoplasmic reticulum stress and improve protein secretion. Proceedings of the National Academy of Sciences 105, 18525–18530 (2008). 10.1073/pnas.0809677105 PMC2587584 19011102

[b39] Pagliassotti M. J. Endoplasmic reticulum stress in nonalcoholic fatty liver disease. Annu Rev Nutr 32, 17–33 (2012). 22809102 10.1146/annurev-nutr-071811-150644

[b40] Arumugam S. T. . Modulation of endoplasmic reticulum stress and cardiomyocyte apoptosis by mulberry leaf diet in experimental autoimmune myocarditis rats. J Clin Biochem Nutr 50, 139–144 (2012). 22448095 10.3164/jcbn.11-44 PMC3303476

[b41] Harriman G. . Acetyl-CoA carboxylase inhibition by ND-630 reduces hepatic steatosis, improves insulin sensitivity, and modulates dyslipidemia in rats. Proc Natl Acad Sci U S A 113, E1796–1805 (2016). 26976583 10.1073/pnas.1520686113 PMC4822632

[b42] Wu M. . Antidiabetic and antisteatotic effects of the selective fatty acid synthase (FAS) inhibitor platensimycin in mouse models of diabetes. Proc Natl Acad Sci U S A 108, 5378–5383 (2011). 21389266 10.1073/pnas.1002588108 PMC3069196

[b43] Viollet B. . Targeting the AMPK pathway for the treatment of Type 2 diabetes. Front Biosci (Landmark Ed) 14, 3380–3400 (2009). 19273282 10.2741/3460 PMC2677695

[b44] Gao X. . Carnitine palmitoyltransferase 1A prevents fatty acid-induced adipocyte dysfunction through suppression of c-Jun N-terminal kinase. Biochem J 435, 723–732 (2011). 21348853 10.1042/BJ20101680

[b45] Kim K. H. Regulation of mammalian acetyl-coenzyme A carboxylase. Annu Rev Nutr 17, 77–99 (1997). 9240920 10.1146/annurev.nutr.17.1.77

[b46] Stancu C. & Sima A. Statins: mechanism of action and effects. J Cell Mol Med 5, 378–387 (2001). 12067471 10.1111/j.1582-4934.2001.tb00172.x PMC6740083

[b47] Rocco D. D. . Aerobic exercise improves reverse cholesterol transport in cholesteryl ester transfer protein transgenic mice. Lipids 46, 617–625 (2011). 21479674 10.1007/s11745-011-3555-z

[b48] Ying R. . The combination of L-4F and simvastatin stimulate cholesterol efflux and related proteins expressions to reduce atherosclerotic lesions in apoE knockout mice. Lipids Health Dis 12, 180 (2013). 24314261 10.1186/1476-511X-12-180 PMC3866605

[b49] Pikuleva I. A. Cholesterol-metabolizing cytochromes P450. Drug Metab Dispos 34, 513–520 (2006). 16434543 10.1124/dmd.105.008789

[b50] Myant N. B. & Mitropoulos K. A. Cholesterol 7 alpha-hydroxylase. J Lipid Res 18, 135–153 (1977). 557521

[b51] Yan L. P. . Puerarin decreases serum total cholesterol and enhances thoracic aorta endothelial nitric oxide synthase expression in diet-induced hypercholesterolemic rats. Life Sci 79, 324–330 (2006). 16472823 10.1016/j.lfs.2006.01.016

[b52] Jeong S. C. . The Korean traditional medicine Gyeongshingangjeehwan inhibits obesity through the regulation of leptin and PPARalpha action in OLETF rats. J Ethnopharmcol 119, 245–251 (2008). 10.1016/j.jep.2008.06.037 18674606

[b53] Wysocka A. C. . Prognostic value of paraoxonase 1 in patients undergoing coronary artery bypass grafting surgery. Medical science monitor: Int Med J Exp Clin Res 20, 594–600 (2014). 10.12659/MSM.890025 PMC3989945 24721823

[b54] Zhou J. . Primary study of leptin and human hepatocellular carcinoma *in vitro*. World J Gastroenterol 14, 2900–2904 (2008). 18473418 10.3748/wjg.14.2900 PMC2710735

[b55] Otte C. . Expression of leptin and leptin receptor during the development of liver fibrosis and cirrhosis. Exp Clin Endocrin Diab 112, 10–17 (2004). 10.1055/s-2004-815720 14758566

[b56] Miyamoto L. E. . Leptin activates hepatic 5’-AMP-activated protein kinase through sympathetic nervous system and alpha1-adrenergic receptor: a potential mechanism for improvement of fatty liver in lipodystrophy by leptin. J Biol Chem 287, 40441–40447 (2012). 23024365 10.1074/jbc.M112.384545 PMC3504759

[b57] Zhai X. Y. . The beta-catenin pathway contributes to the effects of leptin on SREBP-1c expression in rat hepatic stellate cells and liver fibrosis. Br J Pharmacol 169, 197–212 (2013). 23347184 10.1111/bph.12114 PMC3632249

[b58] Lee W. H. & Kim S. G. AMPK-dependent metabolic regulation by PPAR agonists. PPAR Res 2010 (2010). 10.1155/2010/549101 PMC2929615 20814441

[b59] Ables G. P. Update on Pparγ and Nonalcoholic Fatty Liver Disease. PPAR Res 2012 (2012). 10.1155/2012/912351 PMC3431124 22966224

[b60] Inoue M. O. . Increased expression of PPARγ in high fat diet-induced liver steatosis in mice. Biochemical and biophysical research communications 336, 215–222 (2005). 16125673 10.1016/j.bbrc.2005.08.070

[b61] Yu S. M. . Adipocyte-specific gene expression and adipogenic steatosis in the mouse liver due to peroxisome proliferator-activated receptor γ1 (PPARγ1) overexpression. J Biol Chem 278, 498–505 (2003). 12401792 10.1074/jbc.M210062200

[b62] Lee Y. J. . Nuclear receptor PPARγ-regulated monoacylglycerol O-acyltransferase 1 (MGAT1) expression is responsible for the lipid accumulation in diet-induced hepatic steatosis. Proc Natl Acad Sci 109, 13656–13661 (2012). 22869740 10.1073/pnas.1203218109 PMC3427113

[b63] Suzuki A. O. . Leptin stimulates fatty acid oxidation and peroxisome proliferator-activated receptor alpha gene expression in mouse C2C12 myoblasts by changing the subcellular localization of the alpha2 form of AMP-activated protein kinase. Mole Cell Biol 27, 4317–4327 (2007). 10.1128/MCB.02222-06 PMC1900064 17420279

[b64] Bonnefont J. P. . Carnitine palmitoyltransferases 1 and 2: biochemical, molecular and medical aspects. Mole Aspects Med 25, 495–520 (2004). 10.1016/j.mam.2004.06.004 15363638

[b65] Yamagishi S. I. . Leptin induces mitochondrial superoxide production and monocyte chemoattractant protein-1 expression in aortic endothelial cells by increasing fatty acid oxidation via protein kinase A. The Journal of biological chemistry 276, 25096–25100 (2001). 11342529 10.1074/jbc.M007383200

[b66] Matsusue K. G. . Hepatic CCAAT/enhancer binding protein α mediates induction of lipogenesis and regulation of glucose homeostasis in leptin-deficient mice. Mol Endocrinol 18, 2751–2764 (2004). 15319454 10.1210/me.2004-0213

[b67] Um M. Y., Moon M. K., Ahn J. H. & Ha T. Y. Coumarin attenuates hepatic steatosis by down-regulating lipogenic gene expression in mice fed a high-fat diet. Brit J Nutr 109, 1590 (2013). 23597175 10.1017/S0007114512005260

[b68] Ishida-Takahashi R. . Rapid inhibition of leptin signaling by glucocorticoids *in vitro* and *in vivo*. J Biol Chem 279, 19658–19664 (2004). 14993217 10.1074/jbc.M310864200

[b69] Patrick L. Nonalcoholic fatty liver disease: relationship to insulin sensitivity and oxidative stress. Treatment approaches using vitamin E, magnesium, and betaine. Altern Med Rev 7, 276–291 (2002). 12197781

[b70] Kim S. J., Bang C. Y., Guo Y. R. & Choung S. Y. Anti-Obesity Effects of Aster spathulifolius Extract in High-Fat Diet-Induced Obese Rats. J Med Food 19, 353–364 (2016). 26908215 10.1089/jmf.2015.3566

[b71] Xi Y. . Baicalin Attenuates High Fat Diet-Induced Obesity and Liver Dysfunction: Dose-Response and Potential Role of CaMKKbeta/AMPK/ACC Pathway. Cell Physiol Biochem 35, 2349–2359 (2015). 25896320 10.1159/000374037

[b72] Yang J. . Corosolic acid inhibits adipose tissue inflammation and ameliorates insulin resistance via AMPK activation in high-fat fed mice. Phytomedicine 23, 181–190 (2016). 26926180 10.1016/j.phymed.2015.12.018

[b73] Kathirvel E. . Betaine improves nonalcoholic fatty liver and associated hepatic insulin resistance: a potential mechanism for hepatoprotection by betaine. Am J Physiol Gastrointest Liver Physiol 299, G1068–1077 (2010). 20724529 10.1152/ajpgi.00249.2010 PMC2993168

[b74] Wan Y., Liu L. Y., Hong Z. F. & Peng J. Ethanol extract of Cirsium japonicum attenuates hepatic lipid accumulation via AMPK activation in human HepG2 cells. Exp Ther Med 8, 79–84 (2014). 24944601 10.3892/etm.2014.1698 PMC4061235

[b75] Folch J. Lees M. & Sloane-Stanley G. H. A simple method for the isolation and purification of total lipids from animal tissues. J Biol Chem 226, 497–509 (1957). 13428781

