# Supplementary material for: Modified SJH alleviates FFAs-induced hepatic steatosis through leptin signaling pathways
Source: Sci Rep. 2017 Mar 30;7:45425. doi: 10.1038/srep45425 (PMC5371820; doi:10.1038/srep45425)
Supplement: Supplementary Data [file srep45425-s1.pdf]

Modified SJH alleviates FFAs-induced hepatic steatosis through leptin signaling pathways

Dong-Woo Lim[1,2], Shambhunath Bose[3], Jing-Hua Wang[2], Han-Seok Choi[4], Young-mi Kim[5], Young-Won Chin[5], Song-Hee Jeon[6], Jai-Eun Kim[1], Hojun Kim[2*]

[1]Department of Pathology, College of Oriental Medicine, Dongguk University, Goyang, Republic of Korea

[2]Departments of Rehabilitation Medicine, College of Oriental Medicine, Dongguk University, Goyang, Republic of Korea

[3]Applied Surface Technology Inc., 11th Floor, Bldg. A, Advance Institutes of Convergence Technology, Suwon, 16229, Republic of Korea

[4]Division of Endocrinology and Metabolism, Department of Internal Medicine, Dongguk University Ilsan Hospital, Goyang, Republic of Korea

[5]College of Pharmacy and BK21 Plus R-Find team, Dongguk University-Seoul, Goyang, Republic of Korea

[6]Research Institute of Biotechnology, Dongguk University, Goyang, Republic of Korea

[**]Corresponding author:

Department of Oriental Rehabilitation Medicine, Dongguk University-Seoul, Graduate

School of Oriental Medicine, 814 Siksa-dong, Goyang, Gyeonggi-do, Republic of Korea

Tel.: + 82 31 961 9111; Fax: + 82 31 961 9009

E-mail address: kimklar@dongguk.ac.kr (H. Kim)

Supplementary method

UPLC-MS analysis of mSJH and identification of probable secondary metabolites

UPLC-MS analysis of mSJH extract was performed using an Acquity™ Ultra Performance LC system (Waters Corp., Milford, MA, USA) equipped with a binary pump, an autosampler/injector, a column oven, and a UV/visible detector. The UPLC system was coupled with an ACQUITY SYNAPT mass spectrometer (Waters Corp., Manchester, UK) via an electrospray ionization (ESI) interface. Both mSJH and its component extracts were analyzed individually in order to achieve precise fingerprinting of the molecular composition of this herbal formulation.

All extracted samples were dissolved in Dulbecco's phosphate-buffered saline(DPBS**)** at a final concentration of 10 mg/ml. After filtering through a 0.45 mm membrane syringe filter (Sartorius AG, Goettingen, Germany), 0.6 μl aliquots of the samples were injected into the UPLC system via an autosampler. The gradient separation of samples was performed using an ACQUITY UPLC ® BEH C18 column (1.7μm, 2.1 x 50 mm, Waters Corp., Milford, USA) at 30℃. The mobile phase was comprised of 0.1% aqueous solution of formic acid (Merck, Darmstadt, Germany) (A) and 99.9% acetonitrile (B). The initial mobile phase composition

was 90% of A and maintained for 1 min. The mobile phase B was then increased linearly to 100% for 8 min and sustained at 100% for 1.5 min. The linear gradient was then dropped from 100% to 10% of B for 0.5 min and maintained for 4 min. The elution was performed at a flow rate of 0.4 ml/min and the detection was performed at 238 nm. For mass analysis, the temperature of the ion source of MS was maintained at 100℃ and the desolvation temperature was set at 350℃. The flow rates of the cone and desolvation gas (nitrogen) were set at 30 and 600 L h^{-1}, respectively. The capillary voltage used for the analysis of samples was set at 2.0 kV. The fragmentation method was based on collision-induced decomposition and the collision energy was set at 6 eV. All data collected in centroid mode were acquired using the Masslynx NT4.1 software (Waters Corp., Milford, USA).

Cell viability assay

Cell viability was determined using an EZ-Cytox cell viability assay kit (Daeil Lab Service Co, Seoul, Republic of Korea) as per the protocol of the kit manufacturer. Briefly, following the desired treatments of cells grown on a 24-well plate, 10 µl of EZ-Cytox reagent was added to each well. The plates were incubated at 37℃ in a humidified CO_2 incubator for 2 h. Optical density (OD) was then measured at a wavelength of 450 nm using a microplate reader (VersaMax, Molecular Devices, CA, USA).

Determination of tannin content

Tannin content of mSJH was measured following Folin-Denis method [1]. Briefly, 50 µl of the extract was diluted to 7.5 ml by adding distilled water (DDW). To this 0.5 ml of Folin Denis

reagent (Sigma-Aldrich) and 1 ml of sodium carbonate (Na_2CO_3) were added and mixed thoroughly. The volume was then adjusted to 10 ml by adding DDW. The OD was recorded at 700 nm on a microplate reader (Versamax, Molecular Devices, USA). Tannic acid and DDW were used as standard and blank, respectively. Total tannin content is expressed as mg/g of the extract.

Determination of phenol content

Total phenol content of mSJH was measured following folin-ciocalteu method as described previously[2]. Forty microliter aliquot of mSJH was added to 200 μl of folin-ciocalteu reagent (Sigma-Aldrich, St. Louis, MO, USA) in 1160 μl of DDW and mixed thoroughly. The mixture was incubated for 3 min at room temperature and to this 600 μl of 2% sodium carbonate (Na_2CO_3) was added. After 2 h of incubation in dark, the mixture was aliquoted into 96 well plate and absorption was recorded at 765 nm on a microplate reader (Versamax, Molecular Devices, USA). Gallic acid and DDW were used as standard and blank, respectively. Total phenol content is expressed as mg/g of the extract.

Determination of flavonoid content

Total flavonoid content of mSJH was measured using aluminium chloride colorimetric method [3]. Briefly, to 1 ml sample, DDW or aqueous solution of quercetin (Sigma-Aldrich) was added to make final 4 ml volume in a 10 ml volumetric flask as blank and standard, respectively. At time zero, 0.3 ml of 5% sodium nitrite ($NaNO_2$) was added to the flask and

mixed thoroughly. After 5 min, 0.3 ml of aluminium chloride ($AlCl_3$) (10% dissolved in DDW) was added and mixed vigorously. At 6 min, 2 ml of 1 M NaOH was added and the mixture was immediately diluted with 2.4 ml of DDW. Absorbance of the mixture was read at 510 nm on a microplate reader (VersaMax, Molecular Devices, USA) and the total flavonoid content is expressed as mg/g of the extract.

Determination of *in vitro* nitric oxide (NO) production

Following the termination of desired treatments of HepG2 cells in 6-well plates, the culture media was collected from the wells and the content of NO was measured using Griess reagent [0.1% N-(1-naphthyl) ethylenediamine dihydrochloride and 1% sulfanilic acid in 5% phosphoric acid]. Briefly, 100 μl of medium was mixed with 100 μl of Griess reagent in 96 well plates and incubated at room temperature for 10 min. The absorbance was recorded at 540 nm on a microplate reader (VersaMax, Molecular Devices, USA). The nitrite concentration of the samples was determined from a freshly prepared sodium nitrite standard curve.

2,2-diphenyl-1-picrylhydrazyl (DPPH) assay

To determine the free radical scavenging activity of mSJH, 40 μl of various concentrations of this extract was added to 760 μl ethanolic solution of 0.3 mM DPPH. An equal amount of ethanol without sample and DPPH served as control. After 30 min incubation in the dark, absorbance was recorded at 517 nm on a microplate reader (VersaMax, Molecular Devices,

USA). The experiment was performed in triplicate and the percentage of radical scavenging was calculated.

Determination of intracellular Reactive oxygen species (ROS)

Intracellular ROS generation was determined using a Muse® Oxidative Stress kit (EMD Millipore Corporation, Billerica, MA, USA) according to the kit manufacturer's protocols. Briefly, HepG2 cells grown on 24-well plates were incubated without or with different concentrations of mSJH in FBS-free DMEM for 1 h. Following this, H_2O_2 was added to the medium at a final concentration of 0.5 or 1 mM and incubated for 1.5 h to induce oxidative stress. Cells were then washed, trypsinized and harvested and finally treated with reagent based on dihydroethidium for 30 min and analyzed within 1 h using a Muse® Cell Analyzer (EMD Millipore Corporation, USA).

Assessment of bioactivity of single compound derived from mSJH

Several compunds were identified and kindly provided from professor Young-Won Chin (College of Pharmacology, Dongguk University) for assessment of bioactivity on HS. We used same protocols as described in methods at Oil Red O staining and Western blotting part.

Supplementary Table and Figure legends

Table. S1. Human and mice primers used in real-time quantitative PCR.

Fig. S1. UPLC-TOF/MS spectra of (A) mSJH, (B) Morus Fructus, (C) Lycii Radicis Cortex, and (D) Atractylodes Rhizoma preparations. The major compounds of the herbs tentatively identified are indicated by arrows.

Fig. S2. Effect of mSJH on the cell viability of uninduced HepG2 cells (A) and FFAs-induced steatosis model of HepG2 cells (B). Data are expressed as the mean ± SD (n = 3). No statistically significant change in cell viability was found in normal vs mSJH-treated and FFAs- vs FFAs + mSJH-treated cells.

Fig. S3. Radical scavenging activity (A), ROS scavenging properties (B) and inhibitory effect on FFAs-induced nitric oxide production (C) of mSJH. The content of major antioxidants of mSJH is shown (D). Data are expressed as mean ± SD (n = 3). # $p < 0.05$ vs normal and * $p < 0.05$ vs FFAs treatment.

Fig. S4. . Immunoblot images showing the impact of Dexamethasone on leptin protein expression in FFAs-treated HepG2 cells (A) and FFAs-treated HepG2 cells with mSJH (B).

Fig. S5. Effect of mSJH on protein expression levels related with lipid metabolism in HFD-fed mice liver. Relative band intensity was analyzed as compared to β-actin (A) and non-

phosphorylated form (B). Quantified results are expressed as numerical fold change to ND group below the image.

Fig. S6. Effect of major compound contained in mSJH on lipid diposition in FFAs-treated HepG2 cells and notable genes related with lipid regulation. (A) Betaine effect on HepG2 cell viability, (B) Betaine effect on intracellular lipid deposition, (C) Betaine and mSJH effect on genes regulating lipid homeostasis

Table S1 Human and mice primers used in real-time quantitative PCR

Human Primers		
Primer	Forward Primer	Reverse Primer
Leptin	TCCCCTCTTGACCCATCTC	GGGAACCTTGTTCTGGTCAT
β-Actin	TCCCTGGAGAAGAGCTACGA	AGCACTGTGTTGGCGTACAG
Mouse Primers		
Primer	Forward Primer	Reverse Primer
FAS	GCCTCCGTGGACCTTATC	ACAGACACCTTCCCGTCA
ACC	CCAGACCCTTTCTTCAGC	TTGTCGTAGTGGCCGTTC
CPT1α	CGCACGGAAGGAAAATGG	TGTGCCCAATATTCCTGG
AMPKα	AAGCCGACCCAATGACATCA	CTTCCTTCGTACACGCAAAT
LDLR	GAACTCAGGGCCTCTGTCTG	CAGGCTGGATGTCTCTGTGA
SR-B1	GGGCTCGATATTGATGGAGA	GGAAGCATGTCTGGGAGGTA
APOA	AATGTGTATGTGGATGCGGTCAA	AGCCGTTCCTGCAGCTGACTA
ABCA	CGTTTCCGGGAAGTGTCCTA	GCTAGAGATGACAAGGAGGATGGA
CYP7A1	GCTAAGACGCACCTCGTGAT	AGGGCTCCTGATCATTTGAA
CYP8B1	GACCACAGGATGATCCACTTAGC	ACCTTTAGGCCCTAGCATCAC
CYP27A1	CACTTCCTGCTGACCAATGA	CAGTGTGTTGGATGTCGTGTC
β-Actin	GCAAGTGCTTCTAGGCGG	AAGAAAGGGTGTAAAACG

Fig S1 UPLC-TOF/MS spectra

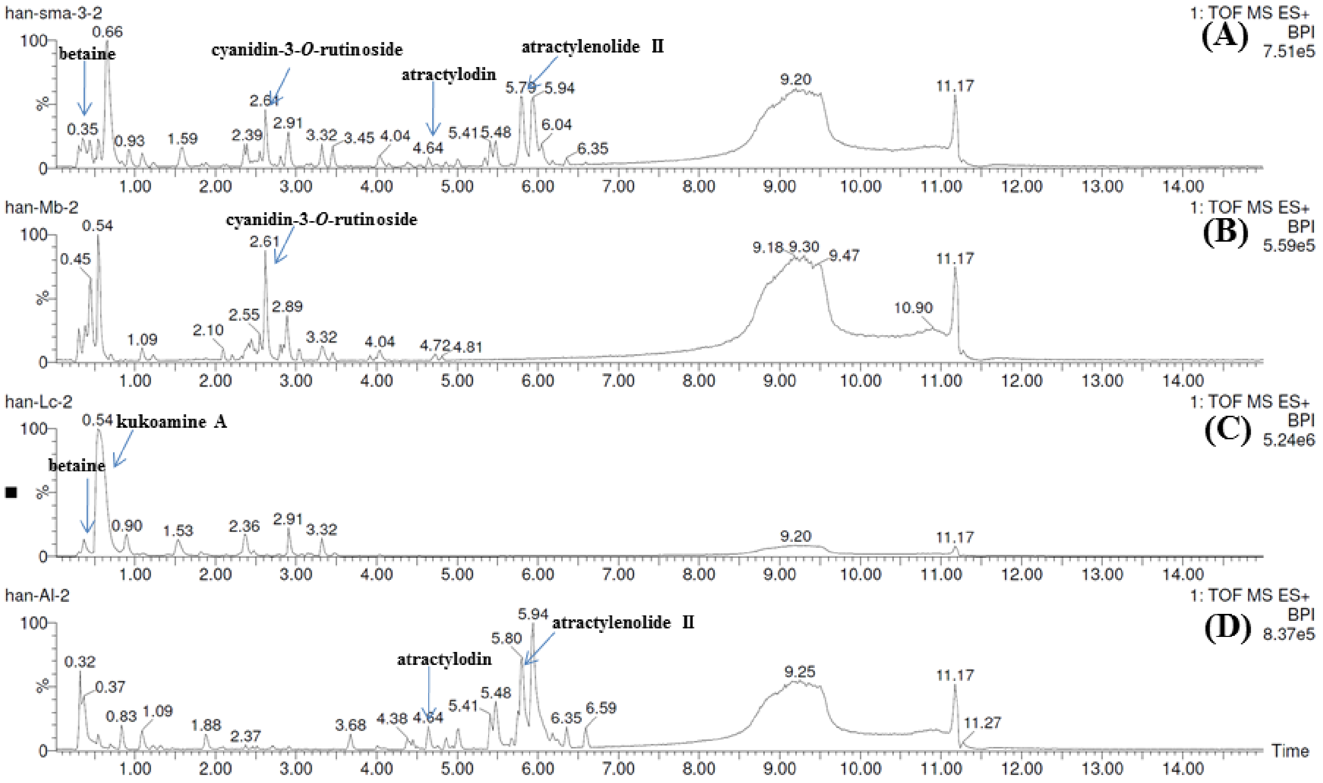

Fig. S2. Effect of mSJH on the cell viability

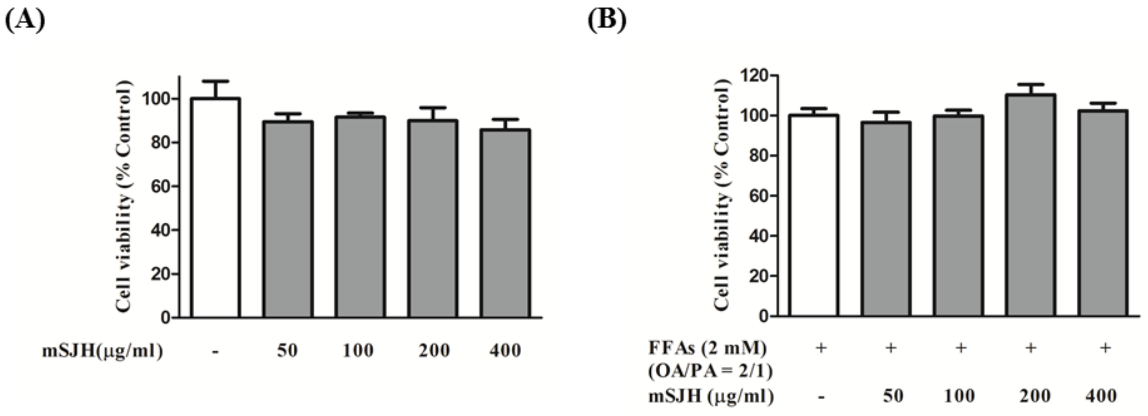

Fig. S3. Radical scavenging activity, ROS scavenging properties and inhibitory effect on FFAs-induced nitric oxide production of mSJH.

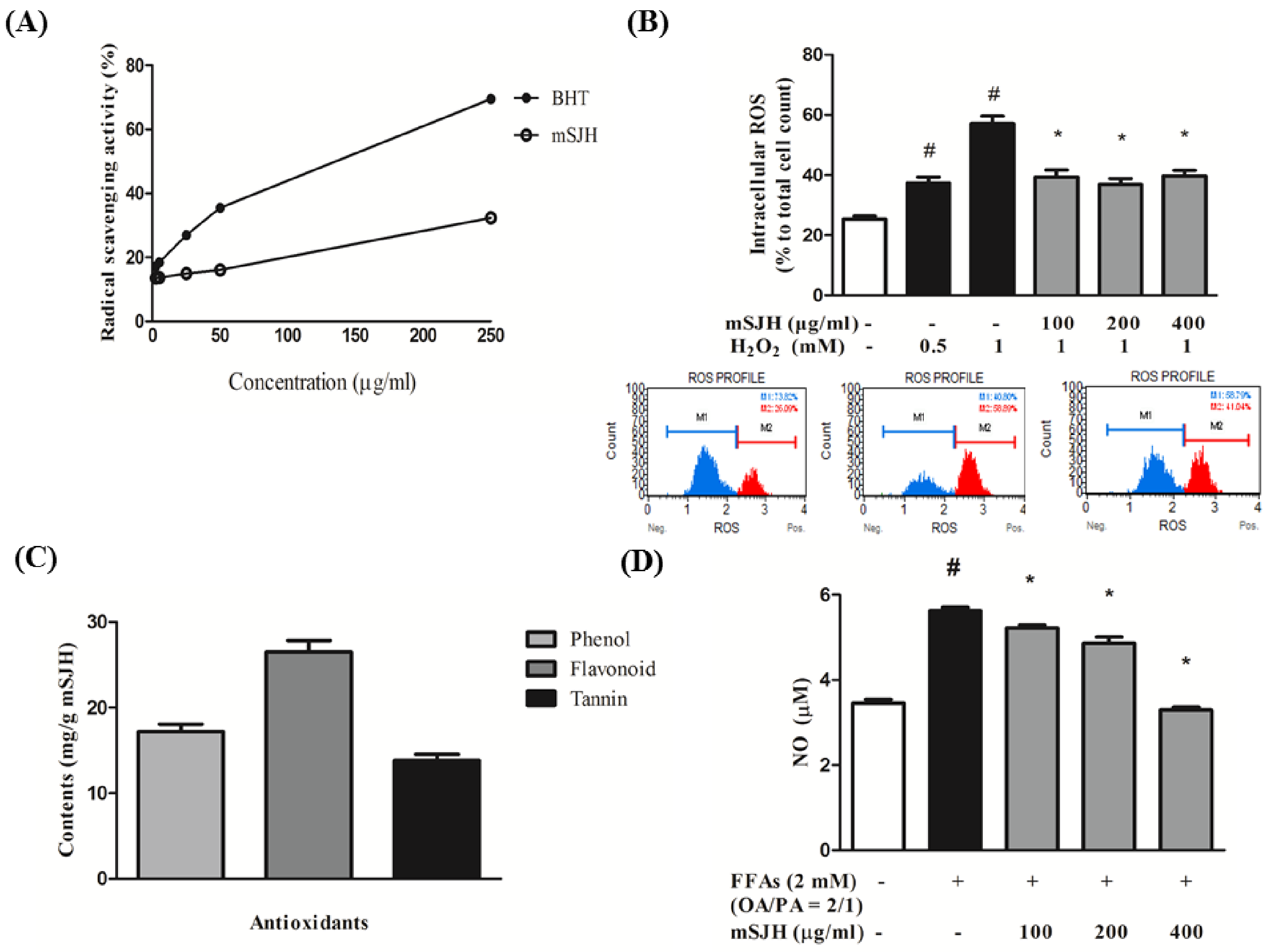

Fig. S4. Impact of Dexamethasone on leptin protein expression

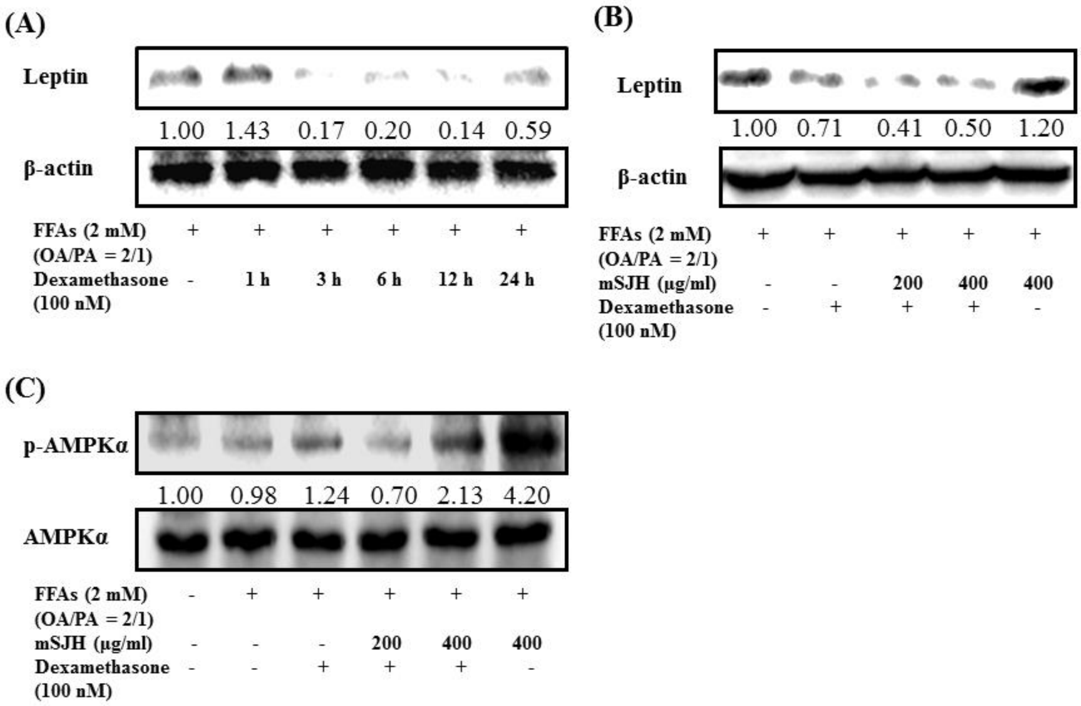

Fig. S5. Effect of mSJH on protein expression levels related with lipid metabolism in HFD-fed mice liver.

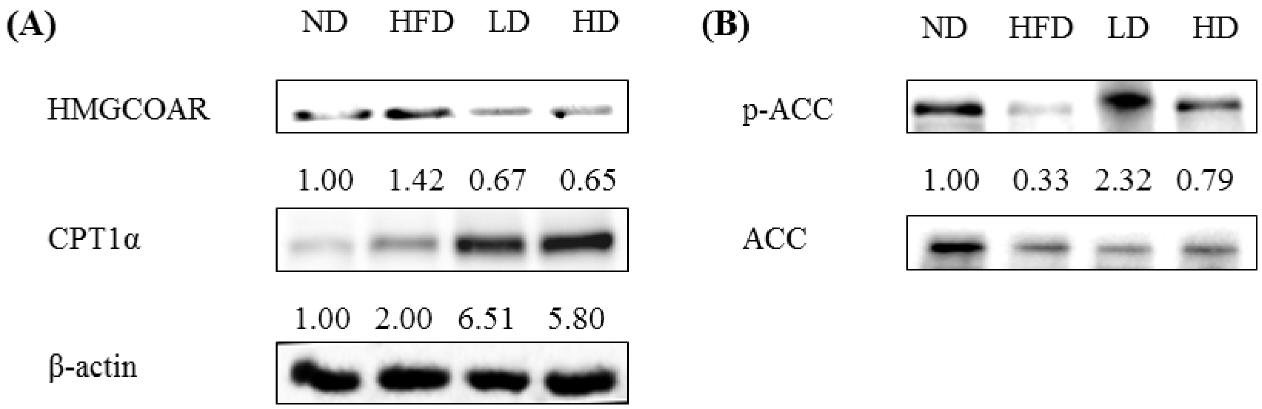

Fig. S6. Effect of major compound contained in mSJH on lipid deposition in FFAs-treated HepG2 cells and notable genes related with lipid regulation.

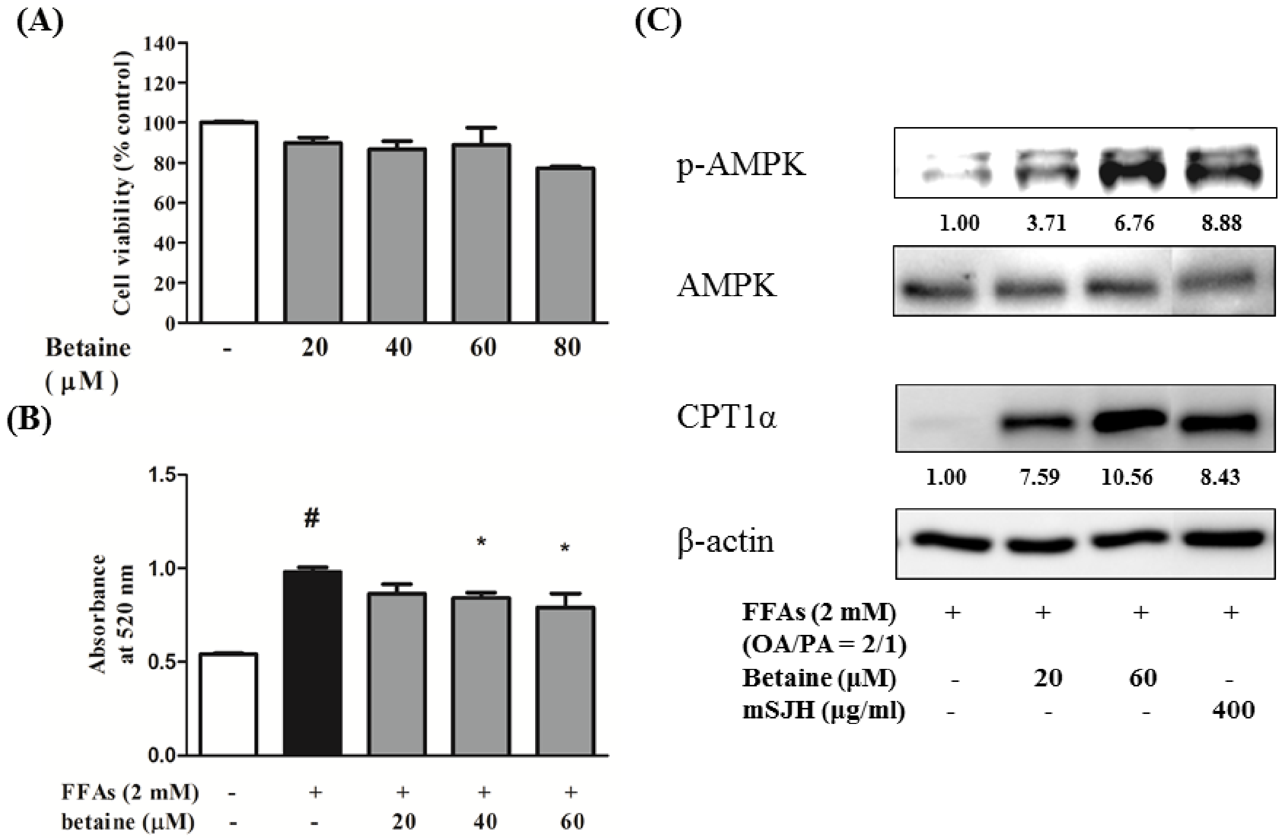

References

1. Polshettiwar S, Ganjiwale R, Wadher S, Yeole P. Spectrophotometric estimation of total tannins in some ayurvedic eye drops. *Indian Journal of Pharmaceutical Sciences* **69**, 574 (2007).

2. Lacikova L, Muselik J, Masterova I, Grancai D. Antioxidant activity and total phenols in different extracts of four Staphylea L. Species. *Molecules* **12**, 98-102 (2007).

3. Zhishen J, Mengcheng T, Jianming W. The determination of flavonoid contents in mulberry and their scavenging effects on superoxide radicals. *Food chemistry* **64**, 555-559 (1999).
